# X-Ray Crystal Structure and Properties of Phanta, a Weakly Fluorescent Photochromic GFP-Like Protein

**DOI:** 10.1371/journal.pone.0123338

**Published:** 2015-04-29

**Authors:** Craig Don Paul, Daouda A. K. Traore, Seth Olsen, Rodney J. Devenish, Devin W. Close, Toby D. M. Bell, Andrew Bradbury, Matthew C. J. Wilce, Mark Prescott

**Affiliations:** 1 Department of Neuro- and Sensory Physiology, University Medicine, Göttingen, 37073, Göttingen, Germany; 2 Department of Biochemistry and Molecular Biology, School of Biomedical Sciences, Monash University, Clayton campus, Victoria, 3800, Australia; 3 School of Chemistry, Monash University, Clayton campus, Victoria, 3800, Australia; 4 Bioscience Division, Los Alamos National Laboratory, Los Alamos, NM, 87545, United States of America; 5 School of Mathematics and Physics, The University of Queensland, Brisbane, Queensland, 4072, Australia; Cardiff University, UNITED KINGDOM

## Abstract

Phanta is a reversibly photoswitching chromoprotein (Φ_F_, 0.003), useful for pcFRET, that was isolated from a mutagenesis screen of the bright green fluorescent eCGP123 (Φ_F_, 0.8). We have investigated the contribution of substitutions at positions His193, Thr69 and Gln62, individually and in combination, to the optical properties of Phanta. Single amino acid substitutions at position 193 resulted in proteins with very low Φ_F_, indicating the importance of this position in controlling the fluorescence efficiency of the variant proteins. The substitution Thr69Val in Phanta was important for supressing the formation of a protonated chromophore species observed in some His193 substituted variants, whereas the substitution Gln62Met did not significantly contribute to the useful optical properties of Phanta. X-ray crystal structures for Phanta (2.3 Å), eCGP123^T69V^ (2.0 Å) and eCGP123^H193Q^ (2.2 Å) in their non-photoswitched state were determined, revealing the presence of a *cis*-coplanar chromophore. We conclude that changes in the hydrogen-bonding network supporting the *cis*-chromophore, and its contacts with the surrounding protein matrix, are responsible for the low fluorescence emission of eCGP123 variants containing a His193 substitution.

## Introduction

GFP-like proteins are valuable tools used extensively in molecular cell biology research [1–5]. In these genetically encoded probes, a tripeptide sequence undergoes a series of post-translational modifications to form a chromophore shielded from bulk solvent within a characteristic 11-stranded β-barrel [6]. Representative members of this protein family have been isolated from a range of marine organisms, and subsequently engineered to generate a large family of proteins with optical properties useful for a wide range of applications. For example, much emphasis has been placed on developing probes with different colour emissions, in particular those with significant fluorescence emission in the near infra-red region that are useful for imaging in living tissues and organisms [7]. Proteins are now available with other optimised properties including increased photostability [8], large Stokes shifts [9], improved quantum yield (Φ_F)_ and brightness [10]. On the other hand, properties considered undesirable, including slow chromophore maturation and environmental sensitivity of fluorescence emission, have inspired the development of useful probes including fluorescent timers [11] and biosensors for pH [12].

A number of naturally occurring weakly fluorescent GFP-like proteins, or chromoproteins, have been reported, and include Rtms5 [13], Rtms1 [14], gtCP [15], cgCP and hcCP [16]. Weakly fluorescent chromoproteins have been usefully imaged using stimulated emission microscopy [17], and have proved useful as a starting point for engineering probes with far-red shifted fluorescence emissions [16,18]. However, the potential to exploit the weakly fluorescent but strong light absorbing properties of these proteins has been largely overlooked. More recently the development of chromoproteins as acceptors for Förster resonance energy transfer (FRET) has been reported. Ultramarine, a monomeric form of the obligate tetramer Rtms5, retains a high molar extinction coefficient and low Φ_F_, making it suitable as an acceptor to a number of different donor fluorescent proteins [19]. The initially bright yellow fluorescent protein was converted to the non-fluorescent REACh series of FRET acceptors using a different approach to engineer useful non-fluorescent tools [20,21]. The fluorescent DsRed (Φ_F_, 0.70) was converted to the chromoprotein DsRed-NF (Φ_F_, <0.001) by introducing four amino acid substitutions [22]. Most recently Phanta, an orange weakly fluorescent photoswitching protein was reported to have utility as a photochromic FRET (pcFRET) acceptor for EGFP, thereby overcoming the requirement for expensive time-resolved fluorescence imaging instrumentation when using non-fluorescent acceptors [5].

Phanta (λ_max_^em^, 516 nm; Φ_F_, 0.003) was isolated from a semi-random mutagenesis screen of the bright green fluorescent reversible negatively photoswitching eCGP123 (λ_max_^abs^, 495 nm; Φ_F_, 0.8; λ_max_^em^, 505 nm), and contains the substitutions His193Gln, Thr69Val and Gln62Met [5]. Phanta can be reversibly photoswitched between two absorbing states corresponding to an anionic (λ_max_^abs^, 506 nm) and protonated (λ_max_^abs^, 387 nm) chromophore, referred to as the ON and OFF states, respectively each of which are weakly fluorescent.

eCGP123 is a bright green FP engineered for extreme stability, and was developed using a two-step process [23]. Using the green fluorescent protein monomeric Azami-Green [24] as a guide, a consensus green protein (CGP) was designed by aligning the amino-acid sequences of 31 different fluorescent proteins [25]. Subsequently, CGP was subjected to an iterative program of evolution, resulting in an extremely thermostable variant eCGP123, the fluorescence emission properties of which remain intact after exposure to high temperature for extended periods. Compared with both mAG and CGP, which became nonfluorescent after 14 h incubation at 353 K, eCGP123 retained 80% of its original fluorescence emission [23]. Unlike mAG, the protein that was used to guide the consensus engineering, eCGP123 possesses useful reversible photoswitching capabilities similar to those of Dronpa. The amino acid sequence of Phanta and eCGP123 are shown aligned to several other photoswitching fluorescent proteins including Dronpa, mEOS and Dendra2 (additional Information S1 Fig) [26,27].

Using site directed mutagenesis and X-ray crystallography, we have investigated the reasons for the very low fluorescence efficiency of Phanta in the ON state. We show that single substitutions at position 193 alone can lead to a dramatic reduction in Φ_F_. Crystal structures indicate that significant alterations to the hydrogen-bonding network or scaffold lying beneath the plane of the *cis*-chromophore and *cis*-coplanar chromophore in Phanta and eCGP^H193Q^, respectively. Reference to a chemical model for photoswitching suggests changes to the protein matrix in these proteins lifts restrictions on the anionic chromophore present in eCGP123 and eCGP123^T69V^ providing access to a phenoxy-twisted intramolecular charge-transfer channel allowing non-radiative decay.

## Materials and Methods

### Mutagenesis, Protein Expression and Purification

Expression vectors encoding eCGP123 variants with a C-terminal 6 x His tag were constructed by site-directed mutagenesis (QuickChange, Invitrogen) using the plasmid pETCK3:eCGP123 [28] as DNA template. Phanta with a C-terminal 6 x His tag was encoded in the vector pET28b:Phanta [5].

Proteins were expressed in the NovaBlue (λDE3) strain of *E*. *coli* and purified by Ni-NTA chromatography as described [28] and appeared as single polypeptides on Coomassie stained SDS-PAGE gels. For crystallisation purposes proteins were subjected to chromatography on a S200 size exclusion column equilibrated in 20 mM Tris-HCl pH 8.0, 300 mM NaCl. Fractions containing variant proteins were pooled and concentrated to 15 mg.ml^-1^ in preparation for crystallisation trials using the hanging drop vapour diffusion technique.

### Spectrophotometry

Absorbance spectra were determined using a Varian Cary 50 spectrophotometer (Agilent Technologies). For pH titrations, proteins in 20 mM Tris-HCl (pH 8.0) were diluted (~ 100-fold) as required into selected 0.1 M buffers. Absorbance spectra were recorded at 25°C 30 s after mixing. Sample pH was monitored using a micro-pH probe. Fluorescence spectra were determined using a Varian Eclipse fluorescence spectrophotometer (Agilent Technologies). All spectra were corrected and were determined under illumination conditions that did not induce photoswitching.

Φ_F_ values were determined for proteins in 20 mM Tris-HCl pH 8.0, 300 mM NaCl at 25°C using solutions of Rhodamine 6G (Φ_F_, 0.95) in ethanol as a standard [13,29].

### Photoswitching of isolated proteins

Photoswitching experiments were routinely performed using protein solutions (150 μl; OD = 1.0 at λ_abs_^max^ in the ON state) in 20 mM Tris-HCl pH 8.0, 300 mM NaCl contained in a single well of a clear 96-well plate [5]. Illumination was performed at 25°C on a plain white surface using light from a cyan Luxeon Rebel LED (λ_peak_, 505 nm; 130 lm; 0.73 mW/mm^2^) or a 5 mm diameter violet LED (λ_peak_, 405 nm; 0.013 lm; 7.8 μW/cm^2^). The protein solution was withdrawn after illumination and absorbance determined. Photoswitching depth is defined as the overall % change in the amount of anionic chromophore species (measured at λ_abs_^max^) on exposure to cyan or violet photoswitching light compared to the starting condition of protein not exposed to photoswitching light and stored in the dark for 24 h. Photoswitching rates were determined by fitting the rate of change of absorbance to a pseudo-first order kinetics mode and the rates normalised to those of eCGP123.

### Time-resolved fluorescence measurements

Fluorescence decay histograms were obtained using the method of Time Correlated Single Photon Counting (TCSPC). A picosecond pulsed supercontinuum fiber laser (Fianium, SC 400-4-pp) was used for excitation and provided ~ 40 ps pulses across the visible and near-infrared at 5 MHz repetition rate. Excitation wavelength selection was achieved using a 700 nm short-pass filter and a 10 nm band-pass filter centered at 488 nm (Chroma). Emission from the sample was collected at 90° to excitation and passed through a monochromator (CVI, dk480) and focused onto a fast response avalanche photodiode detector (APD, Id-Quantique, Id-100). Photon emission times were recorded by a photon counting unit (PicoQuant, PicoHarp 300) with start signals provided by a synchronisation output from the excitation laser and stop signals from the APD detector. Decays were recorded with 10,000 counts in the peak channel at a time resolution of 8 ps/channel. The overall instrument response function (IRF) recorded from a scattering solution (dilute milk powder in water) had a full width half maximum (FWHM) of ~ 90 ps. Further set-up and analysis details are available elsewhere [30]. Fluorescence decay times were obtained by fitting the data with a sum of exponential decay functions convolved with the IRF using time resolved fluorescence analysis software [31]. Goodness-of-fit was judged by the chi-squared parameter (χ^2^) and by inspection of the residuals. All measurements were carried out in 1.0 cm path length cuvettes with absorbance of the solutions kept below 0.1.

### Crystallisation and Structural Determination

The crystallisation, X-ray analysis and detailed description of the parent eCGP123 has been reported elsewhere [28,32]. Diffraction quality crystals of eCGP123^T69V^, eCGP123^H193Q^ and Phanta were obtained at 20°C via the hanging drop method. eCGP123^T69V^, eCGP123^H193Q^ and Phanta protein (~ 15 mg.ml^-1^) in 20 mM Tris-HCl pH 8.0, 300 mM NaCl, were separately mixed in equal volumes with the crystallisation solutions 20% (w/v) PEG 3350, 0.15 M MgCl_2_, 0.1 M Tris-HCl pH 7.5; 20% PEG 3350, 0.1 M MgCl_2_, 0.1 M Tris-HCl pH 7.5 and 21% (w/v) PEG 3350, 0.2 M MgCl_2_, 0.1 M BTP pH 7.4, respectively.

eCGP123^T69V^, eCGP123^H193Q^ and Phanta crystals of approximately 0.1–0.2 mm in length were obtained. Crystals were dipped in perfluoropolyether oil (PFO-X175/08, Hampton Research) for 30 s before vitrification in a nitrogen-gas stream maintained at 100 K. Diffraction images were collected on the MX-1 beamline of the Australian Synchrotron.

Data integration for Phanta was carried out using the HKL-2000 software package [33], whilst XDS was used for eCGP123^T69V^ and eCGP123^H193Q^ [34]. Molecular replacement for eCGP123^T69V^, eCGP123^H193Q^ and Phanta was carried out with Phaser included in the CCP4i software suite [35,36] using eCGP123 [28] trimmed of water molecules, substitutions and the chromophore, as the search model.

Restrained refinement of eCGP123^T69V^, eCGP123^H193Q^ and Phanta models was carried out using the program BUSTER [37] with automatic weighting interspersed with rounds of model building in Win*Coot* [38]. TLS refinement was used in the last few rounds of refinement. Models were checked with Molprobity to guide model building [39]. Water molecules were placed into peaks in the *F*_*O*_ *-F*_*C*_ map and kept in the model if they were located within hydrogen-bonding distance of chemically reasonable groups, visible at 3.0 σ map contour level, and possessed a B-factor <80 Å^2^. The monomer library definition and PDB coordinates of the MYG chromophore were created using the CCP4i Monomer Library Sketcher by inputting and then editing the coordinates of the wild-type CRQ chromophore. Validation of the final eCGP123^T69V^, eCGP123^H193Q^ and Phanta models prior to deposition through PDBj ADIT was carried out using Molprobity. The final models of eCGP123^T69V^, eCGP123^H193Q^ and Phanta were refined to *R*_factor_ 24.37% and *R*_free_ 30.77%, *R*_factor_ 21.04% and *R*_free_ 28.05%, *R*_factor_ 22.99% and *R*_free_ 27.88%, respectively. In each case all residues were within the allowed region of the Ramachandran plot. The coordinates and structure factors for eCGP123^T69V^, eCGP123^H193Q^ and Phanta have been deposited in the Protein Data Bank (4PPK, 4PPL and 4PPJ). Data collection, refinement statistics and a detailed analysis of eCGP123 have been reported [28,32]. The coordinates for eCGP123 (Protein Data Bank accession number 4TZG) were used to generate relevant figures.

## Results and Discussion

### The contribution of amino acid substitutions at positions 62, 69 and 193 to the optical properties of Phanta

Phanta was isolated by expression screening of a library targeting amino acid positions 62/63/65/69 and 191/192/193 in the bright green fluorescent protein eCGP123 [5]. Phanta contains the substitutions Gln62Met, Thr69Val and His193Gln (S1 Fig). A preliminary description of Phanta, and its application as a photochromic acceptor in pcFRET has been reported [5]. In order to better understand Phanta we set out to investigate the contribution of each of the three substitutions, Gln62Met, Thr69Val and His193Gln, to its optical properties.

**Fig 1:**
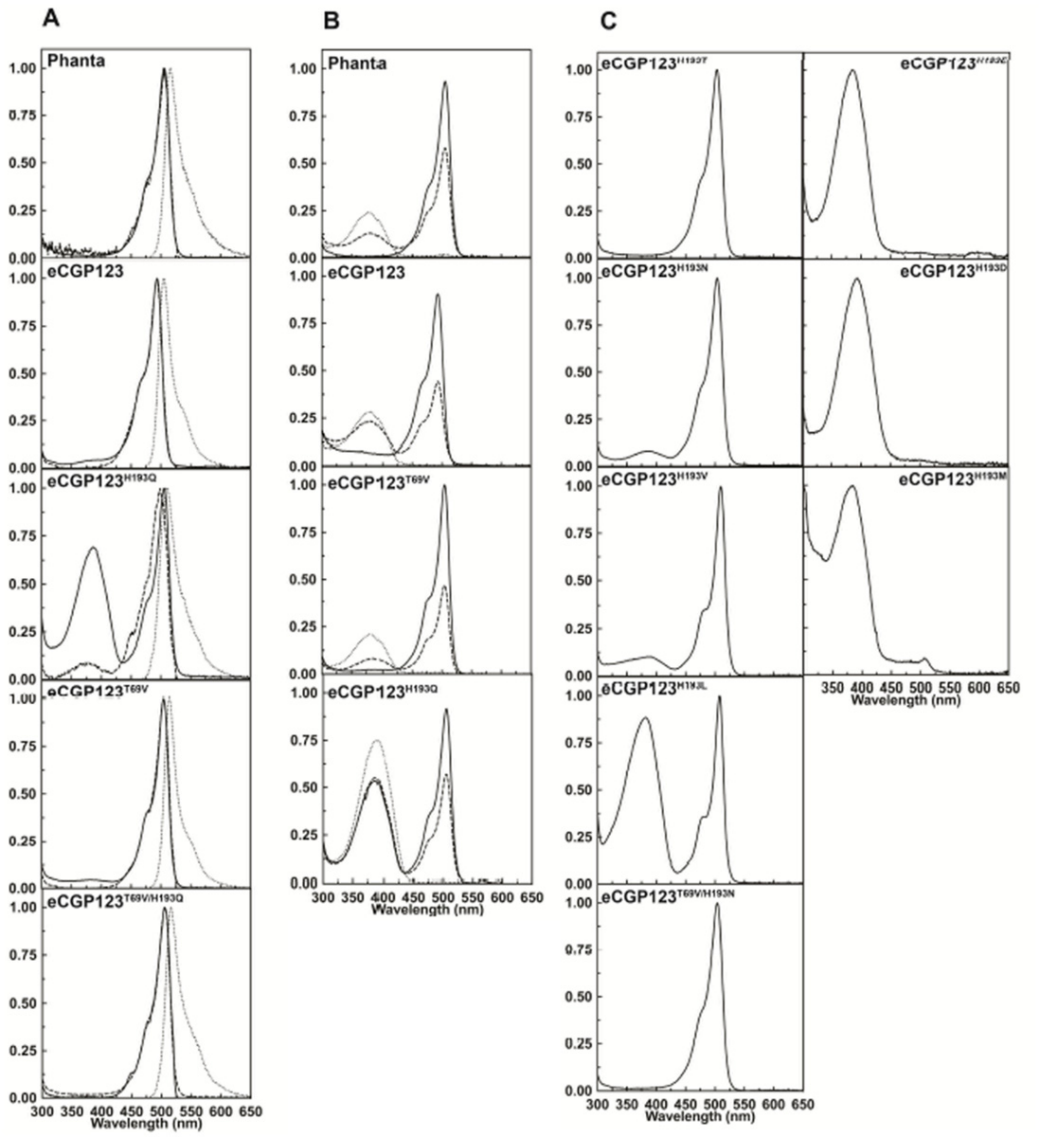
Optical spectra of Phanta and selected variants. (A) Spectra determined at pH 8.0 are shown for Phanta and variants of eCGP123 containing amino acid substitutions that contribute to Phanta. Absorbance (solid line), fluorescence excitation (dashed line) and fluorescence emission (dotted line) are shown. (B) The absorbance spectra are shown for selected variants at pH 8.0 (solid line), pH 6.0 (dashed line) and pH 3.0 (dotted line). (C) Absorbance spectra determined at pH 8.0 are shown for variants of eCGP123 singly substituted at position 193 or doubly substituted at positions 193 and 69.

The singly substituted variants, eCGP123^H193Q^ eCGP123^T69V^, eCGP123^Q62M^ were expressed with a C-terminal 6 x His tag to facilitate protein purification. The absorbance and fluorescence excitation and emission properties were determined at pH 8.0 for each of the purified non-photoswitched proteins (Fig 1 and Table 1). Compared to the parent eCGP123 (λ_max_^abs^, 495 nm), the absorbance spectrum for Phanta (λ_max_^abs^, 506 nm) is significantly red-shifted by 11 nm, resulting in increased spectral overlap with the EGFP emission spectrum [5]. The absorbance spectrum of eCGP123^T69V^ showed a major absorbing species at 506 nm whilst eCGP123^H193Q^ contained significant amounts of a 387 nm species in addition to the 506 nm species. We estimated the proportion anionic and protonated chromophore species in eCGP123^H193Q^ using the molar absorbance extinction coefficients for the fully anionic chomphore of Phanta, and the fully protonated chromophore of eCGP123^H19D^ of eCGP123^H193E^. The fraction of protonated to anionic chromphore CGP123^H193Q^ at pH8.0 was estimated to be 2.4 to 1 suggesting that the majority chromophore species is the protonated state.

**Table 1:** Some optical properties of Phanta, eCGP123 and selected variants.

							^4^Relative photoswitchingdepth (%)		^5^Normalisedphotoswitching rate		
							Photoswitching light				
Protein	^1^λ_max_^abs^ (nm)	λ_max_^ex^ (nm)	λ_max_^em^ (nm)	^2^Φ_F_^ON^	ε @ λ_max_ (M^-1^.cm^-1^)	^3^pK_a_	Cyan (ON→OFF)	Violet (OFF→ON)	Cyan (ON→OFF)	Violet (OFF→ON)	Photoswitching contrast ratio^7^
eCGP123	495	495	505	0.8	60,000@ 495	6	72.5	74.7	1.0	1.0	0.275
Phanta	506	506	516	0.003	98,000@ 505	4	74.8	99.4	1.6	0.82	0.252
^6^Dronpa	503	505	518	0.85	95,000	5.0	95	77	1.7	0.6	0.05
eCGP123H193Q	506 (387)	505 (387/433)	513	0.004	26,500@ 506	6	51.8	66.1	0.9	1.2	0.482
eCGP123T69V	504	506	516	0.32	73,000@ 504	6	91.9	92.5	9.5	1.5	0.081
eCGP123Q62M	493	493	506	0.72	66,000@ 493	-	-	-	-	-	-
eCGP123T69V/H193Q	505	506	518	0.008	78,500@ 505	4	74.0	99.9	1.7	1.1	0.26
eCGP123H193T	505	505	515	0.006	62,000@ 505	INS	61.4	96.7	2.8	1.9	0.386
eCGP123H193N	504/391	506/376	513/410	0.008	72,000@ 504	4	67.1	88.0	1.93	1.8	0.329
eCGP123H193V	510 (390)	510/ (340/420)	520/ (423/525)	0.028	60,000@ 510	-	31.3	89.1	8.25	2.4	0.687
eCGP123H193L	505 (380)	505/ (340)	516 (414)	0.04	15,000@ 505	-	19.1	78.6	2.0	0.8	0.809
eCGP123H193E	385	378 (395)	415/512 (512)	0.003	21,000@ 385	-	-	NP	-	-	-
eCGP123H193D	394	397/495	428/502	0.001	18,500@ 394	-	-	NP	-	-	-
eCGP123H193M	385/510	380/ (380/507)	470 (515)	0.017	3,000@ 385	-	-	-	-	-	-
eCGP123H193F	390	377	475	0.02	1,500@ 390	-	-	-	-	-	-
eCGP123T69V/H193V	504	506	516	0.008	51,000@ 504	4.5	73.1	98.2	2.5	1.9	0.269

^1^ Minor absorbing species are indicated in parentheses.

^2^ Φ_F_ was determined for proteins in the ON state and not exposed to photoswitching light.

^3^ pK_a_’s were determined by titrating absorption of the anionic species at λ_max_^abs^.

^4^ Photoswitching depth is defined as the overall % change in the amount of anionic chromophore species (measured at λ_abs_^max^) on exposure first to cyan (λ_peak_, 505) and then violet (λ_peak_, 405) photoswitching light, compared to the starting condition of protein not exposed to photoswitching light and stored in the dark for 24 h.

^5^ Photoswitching rates are defined as changes in the amount of anionic chromophore species (measured at λ_abs_^max^) and expressed relative for to rate determined for eCGP123. In all cases R^2^ values >98% were obtained.

^6^ Abs/Em/Ex data reproduced from Ando *et al*. 2004.

^7^ Defined as the ratio between amount of anionic state species in the OFF state relative to the ON state.

INS—Proteins showed evidence of precipitation between pH 4–6

NP—Protein did not photoswitch when illuminated with violet light.

We next measured the absorbance spectra of each protein at different pH. As Phanta was incubated at progressively lower pH, amounts of the 506 nm absorbing species decreased whilst amounts of a 387 nm absorbing species increased (Fig 1 and S2 Fig). The 506 and 387 nm absorbing species correspond to the anionic and protonated forms of the 4-hyroxybenzyl chromophore moiety, respectively. eCGP123, eCGP123^T69V^ and eCGP123^H193Q^ showed similar behaviour when pH titrated (Table 1, Fig 1 and S2 Fig). The Phanta chromophore has a pK_a_ of 4 which is significantly lower than that for the other proteins (pK_a_, 6) (Table 1), and is a desirable property that minimises unwanted pH-driven fluctuations when it is used as a pcFRET acceptor [5]. In the case of eCGP123^H193Q^ titration above pH 8 did not diminish the amount of the protonated form, suggesting that two distinct populations of the chromophore exist, only one of which can be titrated for pH. In particular variants of *Aequorea victoria* GFP (*av*GFP), the protonated chromophore is favoured over a wide range of pH due to modification of the hydrogen-bonding network around the chromophore [40]. Recent studies indicate that ground-state proton transfer in green fluorescent proteins can be best described by a two state model in which the chromophore is coupled to a secondary protonation site, most likely a glutamate sidechain [41,42]. It is possible that an equivalent residue (Glu211) plays a role in the protonation behaviour of in eCGP123^H193Q^.

eCGP123 is a bright green fluorescent protein with a high quantum yield (λ_max_^em^, 505 nm; Ф_F_, 0.8), whereas Phanta is weakly fluorescent (Ф_F_, 0.003) with a significantly red-shifted emission spectrum (λ_max_^em^, 516 nm) (Table 1). eCGP123^T69V^ is strongly fluorescent (Ф_F_, 0.32), and like Phanta has a significantly red-shifted fluorescence emission (λ_max_^em^, 516 nm). The properties of eCGP123^Q62M^ are similar to those of eCGP123 suggesting that the Gln62Met substitution does not contribute in a significant way to the properties of Phanta. The properties of a doubly substituted variant, eCGP123^T69V/H193Q^, were found to recapitulate closely those of Phanta (Table 1). Collectively, these data indicate that in Phanta the substitution His193Gln plays a key role in reducing Φ_F_, whilst the substitution Thr69Val is required to suppress formation of the protonated chromophore species observed in eCGP123^H193Q^. Suppression of the formation of the protonated chromophore significantly enhances the extinction coefficient of Phanta at wavelengths where it acts as an acceptor to green emitting FPs.

eCGP123 and Phanta are reversible negatively photoswitching proteins with properties similar to those of the well-characterised bright green photoswitching Dronpa with the principal difference being that the ON state of Phanta lacks significant steady-state fluorescence emission [5,43]. Exposure to photoswitching cyan light drives eCGP123 into a non-fluorescent OFF state that contains a protonated 387 nm absorbing chromophore species, whilst subsequent exposure to violet photoswitching light returns the protein to its emissive ON state containing the anionic chromophore. Phanta, albeit with greatly reduced emissive properties, undergoes similar transitions when exposed to photoswitching light [5].

We investigated the contribution to photoswitching of each of the two individual substitutions His193Gln and Thr69Val. Changes in the absorbance at 506 nm for each variant were recorded, first after exposure to photoswitching light from a cyan LED (λ_peak_, 505 nm) until the rate of photoswitching approached zero, then after photoswitching light from a violet LED (λ_peak_, 405 nm). The relative ON to OFF switching rates of eCGP123^H193Q^ and eCGP123 under violet light illumination were similar (Table 1) but a reduction in amounts of the ON state recovered (~ 66% of starting condition) after exposure to violet light suggested that either a proportion of the chromophore in eCGP123^H193Q^ becomes trapped in the OFF state (Table 1) or is susceptible to photodamage. In order to distinguish between these two possibilities we followed the conversion of eCGP123^H193Q^ from the OFF to ON state at 25°C with or without violet light illumination. The results (S3 Fig) show that under violet light illumination recovery of the ON state for CGP123^H193Q^ driven was minor, whereas thermally induced recovery of the ON state (no illumination) was near complete after 35 mins incubation at 25°C. The conversion OFF to ON rates for Phanta, eCGP123, eCGP123^H193Q^ and eCGP123^T69V^ were determined and are similar (Table 1). These results suggest that photoswitching of eCGP123^H193Q^ into the ON state is inefficient. In contrast, eCGP123^T69V^ underwent very efficient reversible photoswitching (~ 92% photoswitching depth) and had ~ 10 fold increased OFF rate. The substitutions combined in eCGP123^T69V/H193Q^ appear compensatory and confer photoswitching properties similar to those of Phanta (Table 1).

Exposure of eCGP123^H193Q^ to violet photoswitching light did not reduce the amount of the protonated species present at pH 8.0. Compared to the ON state, the λ_max_^abs^ for the OFF state in the protonated species of eCGP123^H193Q^ was red-shifted by ~ 3 nm (data not shown). These results suggest that eCGP123^H193Q^ in the OFF state contains two populations of protonated chromophore in different environments which presumably cannot interconvert. Furthermore, it is possible that the protonated species in eCGP123^H193Q^ and generated at low pH (< pK_a_) corresponds to yet a third chromophore species; the protonated chromophore generated by photoswitching and at low pH exist in different environments.

### X-ray crystal structures of Phanta, eCGP123^T69V^ and eCGP123^H193Q^

We next determined the X-ray crystal structure of Phanta, eCGP123^T69V^ and eCGP123^H193Q^. We refer below to some aspects of an eCGP123 structure, the details of which have been reported [28,32]. Orange crystals of Phanta and eCGP123^H193Q^, and bright green crystals of eCGP123^T69V^ belonged to the space group P 1 2_1_ 1, with four protomers per asymmetric unit. The structures were determined for Phanta, eCGP123^T69V^ and eCGP123^H193Q^ to 2.3 Å, 2.0 Å and 2.2 Å resolution, respectively. The crystal data and refinement statistics are presented in Table 2. The final model for each protein, which consists of four protomers (residues 7–222) and four chromophores, exhibit excellent stereochemistry (Table 2). The rms deviation (for α-carbon atoms) between the four protomers in the asymmetric unit for each protein is less than 0.32 Å. Accordingly structural analysis will, unless otherwise stated, be confined to one protomer. Chromophore contacts for each protein are detailed in S1, S2 and S3 Tables.

**Table 2:** Phanta, eCGP123^T69V^ and eCGP123^H193Q^ data collection and refinement statistics.

Protein	Phanta	eCGP123^T69V^	eCGP123^H193Q^
**Data collection** ^**a**^			
Space group	P 1 2_1_ 1	P 1 2_1_ 1	P 1 2_1_ 1
Unit cell a, b, c (Å)	69.8, 79.4, 71.0,	73.0, 81.3, 74.0	72.6, 83.4, 74.7
Unit cell α, β, γ (°)	90.0, 102.4, 90.0	90.0, 107.6, 90.0	90.0, 102.2, 90.0
Resolution (Å)	34.63–2.30 (2.4–2.3)	50.0–2.0 (2.05–2.0)	50.0–2.2 (2.26–2.2)
Number of unique reflections	33187 (3190)	55029 (4100)	43884 (3246)
Multiplicity	3.8 (3.8)	3.1 (3.0)	5.4 (5.18)
Completeness (%)	98.5 (94.8)	98.6 (99.4)	99.1 (99.95)
I/σ	23.2 (2.6)	12.16 (1.91)	12.9 (3.64)
Rsym (%)	4.7 (54.6)	6.8 (64.8)	9.1 (49.6)
**Refinement**			
*R*_factor_ (%)	22.99	24.37	21.04
*R*_free_ (%)^b^	27.88	30.77	28.05
*r*.*m*.*s*. *deviations*			
Bond lengths	0.018	0.016	0.008
Bond angles	2.471	1.710	1.05
Ramachandran plot			
Most favoured (%)	95.5	91.3	97.4
Additional allowed regions (%)	4.5	8.6	2.6
Outliers (%)	0.0	0.0	0.0
*B*-factors (Å^2^)			
Average chromophore for O^H^	51.16	27.91	50.16
Average side chain	67.71	38.64	60.81

^a^ Values in parentheses refer to the highest resolution shell.

^b^ *R*free was calculated with 5% of the diffraction data selected randomly and excluded from refinement.

**Fig 2:**
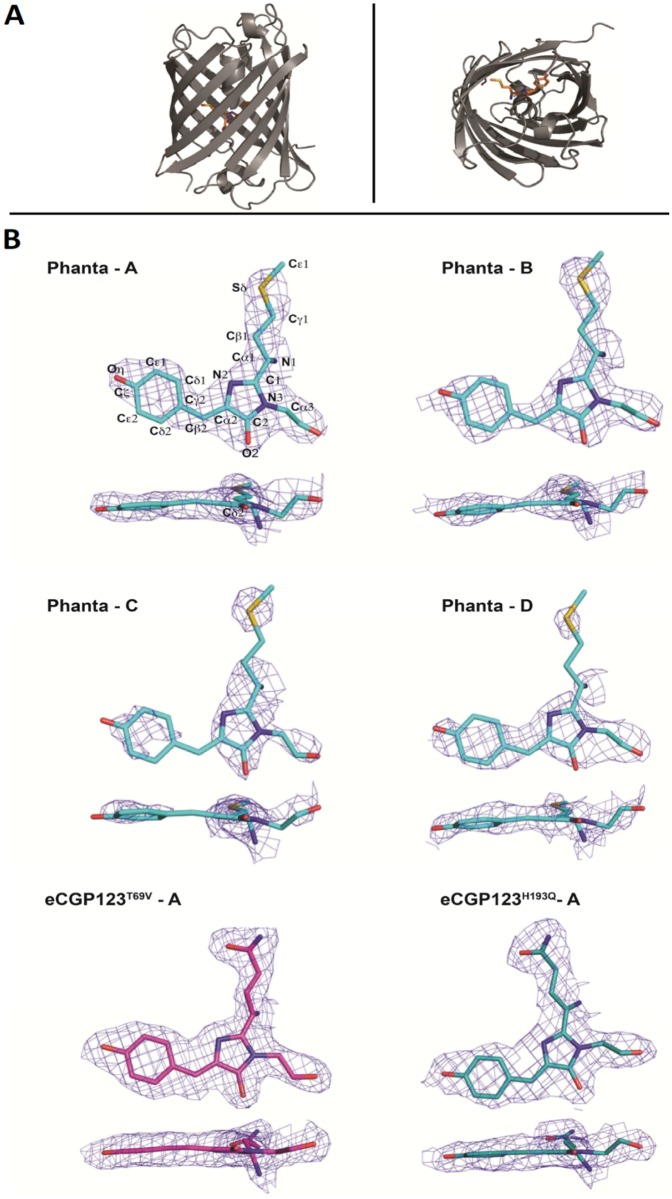
Phanta structure. (A) A schematic ribbon representation of an isolated protomer of Phanta (grey) showing two views (one rotated through 90°) of the 11-stranded β-can motif typical of GFP-like proteins and the central α-helix with the chromophore (orange) represented in stick format. (B) Orthogonal views of the final 2*Fo-Fc* electron density superposed onto the structure for each of the four protomers of Phanta, and protomer A of eCGP123^T69V^ and eCGP123^H193Q^.

The protomers in each protein are typical of the GFP-superfamily and consist of an 11-stranded β-barrel (or β-can) with two small helices forming interconnecting loops protecting the chromophore from bulk solvent. The mature chromophore, consisting of the circularised tripeptide sequence located at the centre of the β-barrel, is linked via two *trans* peptide bonds neighbouring Phe65 and Asn69 to an α-helix running coaxially to the β-can axis (Fig 2). The chromophore tripeptide Met62-Tyr63-Gly64 in Phanta, and Gln62-Tyr63-Gly64 in eCGP123^T69V^ and eCGP123^H193Q^ give rise to the *p*-hydroxybenzylidineimidazolinone chromophore, typical of many members of the GFP superfamily. Since the chromophores in Phanta and eCGP123^T69V^ have a pK_a_ value of 4 and 6 respectively (Table 1), we consider the chromophore to represent the anionic form as crystals were obtained at pH 7.5 or above. The spectral data obtained for eCGP123^H193Q^ suggest the possibility that both the protonated and anionic chromophore are present in the structure but only a single chromophore species appears to be represented in the structural data.

Omit maps show that the electron density corresponding to the imidazolinone moiety of the chromophore in each of the four protomers of Phanta is well defined (Fig 2). The imidazolinone is held rigidly in place by seven hydrogen bonds and nine water-mediated hydrogen bonds (S1 Table). The imidazolinone N2, N3 and O2 each hydrogen bond with the backbone of Pro59. The imidazolinone O2 hydrogen bonds with Arg91 and Asn65, with the latter also hydrogen bonding to imidazolinone N3. In contrast, there is evidence for disorder around the 4-hyroxybenzyl ring in protomers C and D of the Phanta structure, suggesting that it is mobile within the chromophore cavity. Since a rigid chromophore is considered a prerequisite for efficient fluorescence emission [6,44,45], it is possible the increased mobility observed contributes to the low fluorescence efficiency of Phanta (Φ_F_, 0.003). However, electron density corresponding to the 4-hyroxybenzyl ring in protomers of both the weakly fluorescent eCGP123^H193Q^ (Φ_F_, 0.004) and strongly fluorescent eCGP123^T69V^ (Φ_F_, 0.32) is well defined, indicating a rigid chromophore (Fig 2), and suggesting that a mobile chromophore is not responsible for poor fluorescence emission of eCGP123^H193Q^.

In the absence of electron density corresponding to the predicted position for a *trans-* chromophore conformation, the chromophores in Phanta, eCGP123^H193Q^ and the brightly fluorescent eCGP123^T69V^ have been modelled in the *cis*-conformation. A *trans* chromophore conformation has been associated with GFP-like proteins with low fluorescence efficiency such as the pocillioporin Rtms5 [13,18] and the non-fluorescent OFF state of negatively photoswitching proteins such as Dronpa [46]. However, the strongly fluorescent eqFP611 has been shown to contain a *trans-* chromphore [47].

The chromophores in eCGP123^H193Q^ and eCGP123^T69V^ are essentially coplanar with the 4-hydroxybenzyl ring twisted out of plane relative to the imidazolinone ring by only 4.6° and 1.8°, respectively averaged across all four protomers. The poorly defined electron density for Phanta does not allow angles to be determined with any certainty.

**Fig 3:**
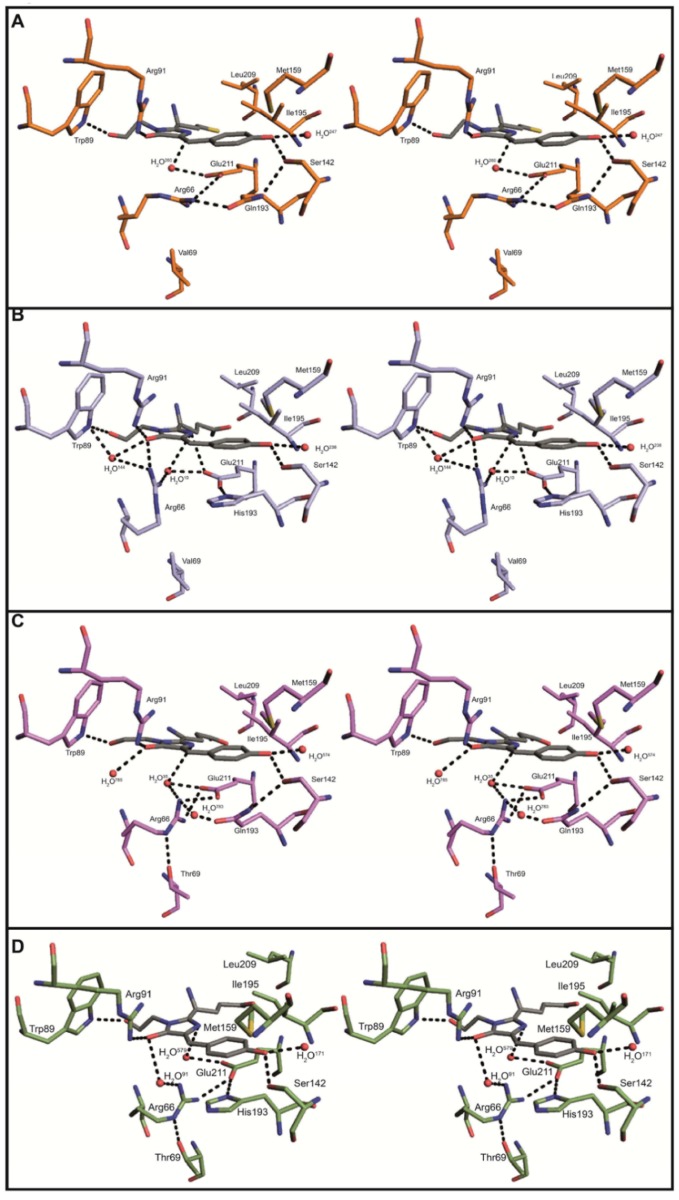
The chromophore environment of Phanta, eCGP123^T69V^, eCGP123^H193Q^ and eCGP123. Stereoviews are shown comparing the chromophore environments and hydrogen bond networks for (A) Phanta; (B) eCGP123^T69V^; (C) eCGP123^H193Q^ and (D) eCGP123. Numbered waters are shown as red spheres.

**Fig 4:**
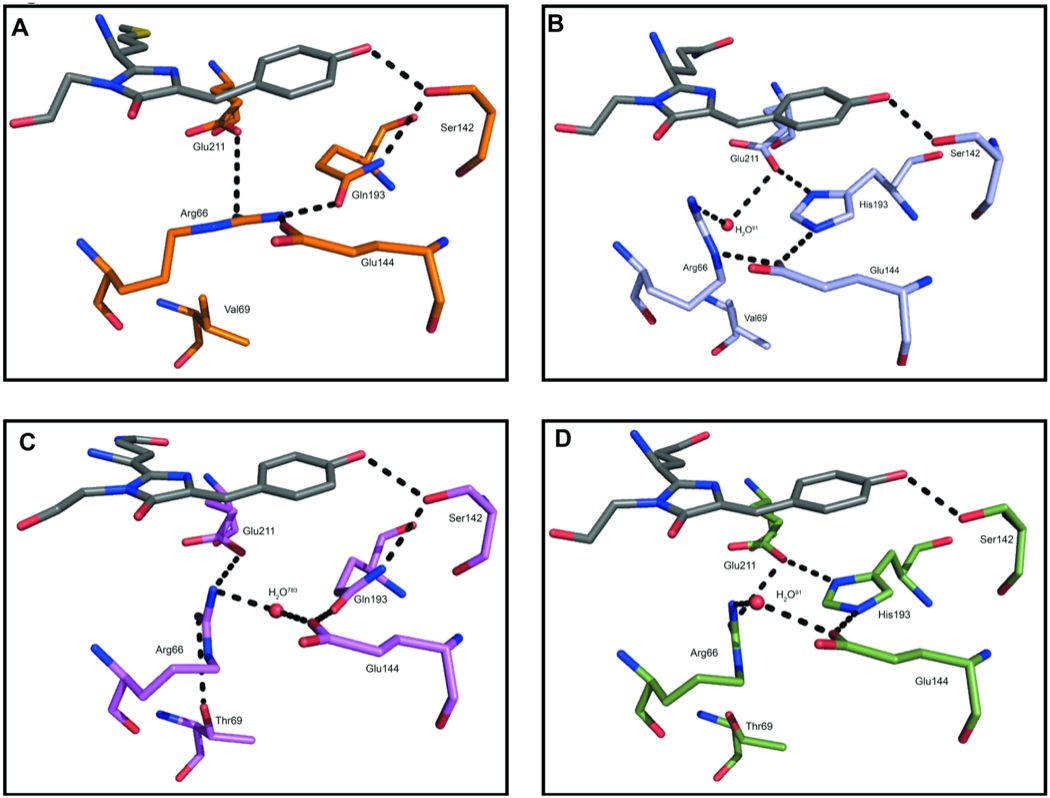
Alterations to the network of interactions that contribute to a scaffold around the chromophore of Phanta, eCGP123^T69V^ and eCGP123^H193Q^ and eCGP123. The network of interactions involving the chromophore and selected side chains differ between the non-fluorescent proteins (A) Phanta and (C) eCGP123^H193Q^, and the highly fluorescent proteins (B) eCGP123^T69V^ and (D) eCGP123. Numbered waters are shown as red spheres.

In eCGP123^T69V^ the imidazole ring of His193 stacks against the 4-hydroxybenzyl ring forming a π-π interaction (Fig 3). Presumably the histidine is charged as it interacts with side chains of the conserved Glu148 and Glu215. In Phanta and eCGP123^H193Q^ the imidazole ring of His193 (Fig 3) is substituted by the polar side chain of a glutamine leaving the chromophore with a reduced number of stabilising interactions (S2 and S3 Tables). In eCGP123^T69V^ and eCGP123 the side chains of His193, Glu215, Glu148, Arg66 and a water (H_2_O^91^) form a planar network of hydrogen bonds and salt bridges beneath the chromophore (Fig 4). Although the Thr69Val substitution results in reorientation of the Arg66 side chain in eCGP123^T69V^, the water (H_2_O^91^) changes position maintaining the network of interactions beneath the chromophore observed in eCGP123. Similar polar networks have been recognised in other highly fluorescent proteins including DsRed [45,48], Dendra2 [26], amFP486 [49] and eqFP611 [47,50]. It is thought such networks restrict chromophore dynamics, thereby contributing to its stability, resulting in a high Φ_F_. Accordingly, in the weakly fluorescent Phanta and eCGP123^H193Q^ the planar network is significantly altered, leaving the chromophore without the underlying scaffold present in eCGP123^T69V^ and eCGP123 (Fig 3 and Fig 4). In Phanta and eCGP123^H193Q^, Glu215 no longer interacts with the side chain of Gln193 that replaces the histidine at position 193. Instead a hydrogen bond is formed between the N^δ1^ of Gln193 and the O^γ^ of Ser142, which in turn forms a hydrogen bond with O^η^ of the chromophore. In eCGP123^H193Q^ contact between Glu144 and Arg66 is maintained through a water-mediated (H_2_O^783^) hydrogen bond. Collectively these data suggest that the very low fluorescence efficiency of Phanta and eCGP123^H193Q^ is a result of removing the stabilising effects of π-π interactions between His193 and the chromophore in the excited state, together with alterations to the scaffold extending around the chromophore.

Fluorescence lifetimes were determined for Phanta and some of its variants (Table 3). Fluorescent decay profiles for both Phanta and eCGP123^H193Q^ were dominated (99 and 90%, respectively) by very short lifetime components, within the FWHM of the IRF and close to the temporal resolution of what can be extracted using deconvolution methods. Values of 22 ps and 13 ps, respectively, were determined for these main decay components for Phanta and eCGP123^H193Q^ and are consistent with the very low quantum yields observed for these two proteins. However, it should be noted that these values have considerable uncertainty and the lifetimes may be shorter still in reality. By comparison, eCGP123 contained a single species decaying with a fluorescence lifetime of 2.3 ns whilst eCGP123^T69V^ contained a major and minor species with a fluorescence lifetime of 1.33 ns and 2.62 ns, respectively. It is possible that the chromophore environment of eCGP123^T69V^ can exist in two emissive conformations although the structural data appears to show evidence of single conformation. These results indicate that the excited state chromophore in Phanta undergoes rapid deactivation through fast internal conversion. The weakly fluorescent REACh (Φ_F_, 0.02) also has a short fluorescence lifetime (~ 320 ps) compared to the parent yellow fluorescent protein (2.9 ns) [20].

**Table 3:** Fluorescence lifetime measurements of Phanta, eCGP123^H193Q^ and eCGP123^T69V^ and eCGP123.

Protein Variant	Fluorescence lifetime (ps)*
Phanta	22 (99.1%)
	170 (0.8%)
	1,670 (0.1%)
eCGP123^H193Q^	13 (90%)
	110 (5.7%)
	1,002 (1.5%)
	2,940 (2.5%)
eCGP123^T69V^	1,330 (30.1%)
	2,620 (69.9%)
eCGP123	4,200 (100%)

* χ^2^ values of 1.16, 1.05, 1.00 and 1.23 were obtained for fitting of data for Phanta, eCGP123^H193Q^, eCGP123^T69V^ and eCGP123, respectively. Proportion of each lifetime is indicated in parentheses. Excitation was provided using a 10 nm band pass filter centred on 488 nm.

Compared to eCGP123, the hydrogen bond network around the chromophore in Phanta and eCGP123^H193Q^ is extended through O^x^ of Ser142 to include the O^η^ of the 4-hydroxybenzyl ring (Fig 3 and Fig 4). Extension of the hydrogen bond network around the chromophore has been reported to be responsible for the red-shifted spectra of other fluorescent proteins including eqFP670 and TagRFP675 [51,52], and presumably contributes to the red-shifted spectra of Phanta and eCGP123^H193Q^ (Table 1). Since the extended hydrogen bond network is absent in eCGP123^T69V^ (λ_max_^abs^, 504 nm) more than one mechanism is likely operating to control the red-shift in these proteins. The aliphatic side-chain of the valine substitution in eCGP123^T69V^ can no longer hydrogen-bond with the N^ε^ of Arg66 allowing the N^NH1/NH2^ of Arg66 to extend towards the imidazolinone O2 forming a direct hydrogen bond and a water (H_2_O^144^) mediated hydrogen-bond (Fig 3). Closer interaction of the Arg66 side chains with the imidazolinone ring would increase charge stabilisation on the ring (Fig 4). Since excitation of the chromophore is understood to result in a shift of electron density from the 4-ydroxybenzylidine to the imidazolinone ring [53], the energy of the excited state would be lowered resulting in a red-shift. It has been reported that the proximity of the charged side chains of arginine or lysine at the same position in other fluorescent proteins such as mKO, Dendra2 and mEos are responsible for contributing to their red-shifted spectra [25, 33].

The Phanta chromophore has a pK_a_ of 4 making its optical properties usefully pH-resistant in live cell imaging applications. In Phanta the hydrogen bond network around the chromophore extends from the O^η^ of the 4-hydroxyphenyl ring to the N2 of the imidazolinone ring (Fig 3), and the O2 of the imidazolinone ring via protein matrix hydrogen bonds involving the side chains of Tyr177, Arg91 and Glu144 (S4 Fig). In eCGP123^T69V^ and eCGP123 the hydrogen bond network from O^η^ of the phenyl ring is truncated and does not extend beyond the O^γ^ of Ser142. In eCGP123^H193Q^ the network extends from the O^η^ of the phenyl ring but terminates at N2 instead of O2 of the imidazolinone ring. Presumably the hydrogen bond network between the O^η^ of the phenyl ring and the O2 imidazolinone lowers the stabilisation of the anionic form of the chromophore. Lack of bonding between the imidazolinone O2 and the side chain of Arg66 has been reported to influence protonation of the 4-hydroxyphenyl ring, and is reported to be responsible for the relatively high pK_a_ for Dendra2 compared to other related proteins [26]. This mechanism is not dominant in the case of Phanta as Arg66 hydrogen bonds to the O2 of the imidazolinone ring in eCGP123^T69V^ (pK_a_, 6), but not Phanta (pK_a_, 4).

### Alternative amino acid substitutions at position 193

Our data indicates that the identity of the amino acid side chain occupying position 193 plays an important role in the fluorescence emission of eCGP123. We next investigated eCGP123 variants each containing an alternate amino acid substitution at position 193. Variants carrying amino acid substitutions with a polar side chain (threonine or asparagine) had low Φ_F_ values that were similar to that of Phanta. In contrast those substitutions with hydrophobic side chains (valine, leucine, methionine and phenylalanine) resulted in Φ_F_ values higher than that observed for Phanta, but well below that for eCGP123 (Fig 1 and Table 1). These results indicate a histidyl ring at position 193, which stacks with the 4-hydroxybenzyl ring in eCGP123^T69V^ (Fig 3), as being important for efficient fluorescence emission. However, hydrophobic side chains may be able to provide some stability to the 4-hydroxybenzyl resulting in Φ_F_ values marginally higher than those of Phanta.

The identity of the amino acid side chain at position 193 also influences the charge state of the chromophore. The anionic chromophore is favoured by the small hydrophobic side chain of valine, and polar side chains of threonine and asparagine. The larger side chains of leucine and methionine, and the larger negatively charged side chains of glutamate and aspartate favour formation of the protonated chromophore (Fig 1 and Table 1). Presumably the negatively charged side chains of aspartate and glutamate in the chromophore environment favour formation of the protonated chromophore. This situation is different to the photoswitched state of Dronpa where the chromophore environment consists of neutral residues providing no possible hydrogen bonding pair and thus, a protonated chromophore is observed.

The photoswitching properties were determined for the different His193 substituted variants (Table 1). When present, the anionic chromophore in each variant underwent reversible photoswitching. Notably the His193Thr and His193Asn substituted variants showed similar photoswitching properties to those of eCGP123 and Phanta. The His193Val and His193Leu substituted variants underwent some photoswitching but with lower efficiency as indicated by the reduced photoswitching depth. The His193Glu and His193Asp substituted variants contained only a protonated chromophore, and did not photoswitch when exposed to violet illumination. These data indicate that photoswitching can be maintained in proteins with a substitution other than Gln or His at position 193, and suggest that eCGP123^H93T^ or eCGP123^H193N^ represent practical alternatives to Phanta. Although many reversible photoswitching fluorescent proteins including KFP1 [54], Dronpa [43], Padron [55], Iris [56] and rsTagRFP [57] have a histidine at the equivalent position of 193 in Phanta, others such as Dreiklang [58] and rsEGFP1 [59] do not.

eCGP123 is a thermally stable protein. It is not clear from our studies what affect increased stability may have on the mechanism of photoswitching. It is conceivable that decreased conformational freedom within the structure of eCGP123 would influence the movement of the chromophore, and its stability in the ON or OFF state. It has been established through NMR studies that photoswitching of Dronpa into the OFF state is accompanied by part of the β-barrel becoming flexible leading to a non-radiative process [60,61]. It remains to be established if such structural flexibility in Dronpa is a property of photoswitching proteins such as eCGP123 that share a similar mechanism.

### Photoswitching without fluorescence emission

**Fig 5:**
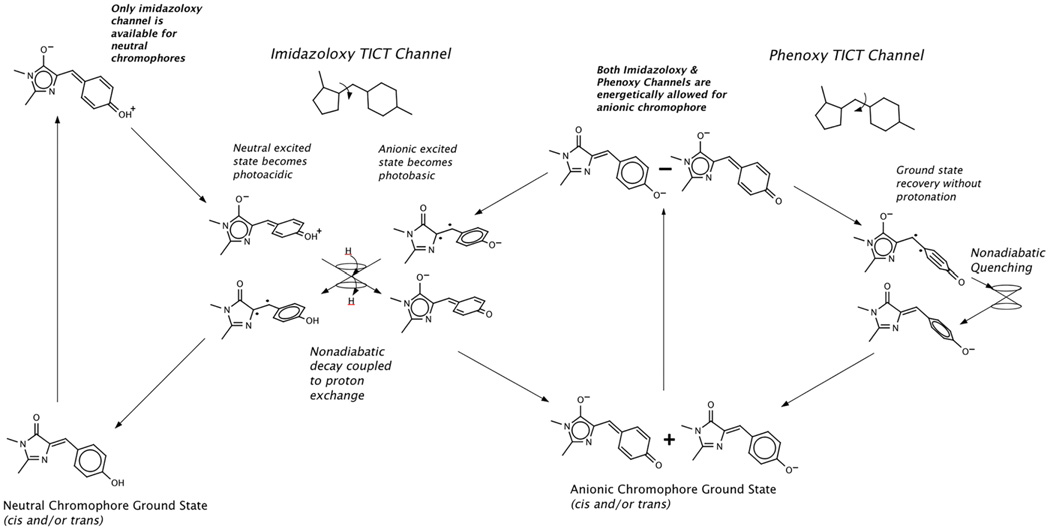
Decay via twisted internal charge-transfer states of the chromophore with mechanism for coupling to proton transfer. A schematic depiction of the hypothetical photoswitching mechanism. The mechanism is a variation of the photoswtiching mechanism for negative-mode reversibly photo switchable fluorescent proteins suggested by Olsen Lamothe & Martínez [62]. The key point is that there are two twisted intramolecular charge-transfer channels available to the anionic chromophore, whose ground state is a quantum superposition of states with distinct bond alternation. The excited state is the anti-phase combination of the same two bonding states. The two channels correspond to the two bonds on the bridge. The neutral chromophore only has one TICT channel, corresponding to the I bond, reflecting the definite bond alternation of its ground state. The neutral excited state has opposing bond alternation and charge localisation, relative to its ground state. If the anionic chromophore accesses the imidazolinone TICT channel, then its excited state becomes photo basic relative to the optically excited state, increasing the probability that it will accept a proton. Acceptance of a proton by the anion TICT state in the imidazolinone channel deactivates the excited state, yielding a neutral chromophore population in its ground state. If the anion accesses the phenoxy TICT channel in its excited state, electron density is pushed off the phenoxy, making it more photo acidic than the optically prepared excited state, and preventing proton uptake. Deactivation of the anionic excited state can still occur through the conical intersections that exist in the phenoxy TICT channel, yielding an anionic ground state. If the combined rate of access of the phenoxy and imidazolinoxy channels is much larger than the rate of fluorescence, then the "ON" state is non-fluorescent, as observed in Phanta. The kinetics of photoswitching will depend on the precise shape of the electronic state surfaces and on the friction experienced by the chromophore during its excited-state dynamics. The coupling between TICT state channels and protonation state can be understood using an adaptation of a two-state electron transfer model [74].

Phanta and a number of the eCGP123^H193X^ variants reported in this study (Table 1) are unique amongst the class of reversible photoswitchable fluorescent proteins (RSFP) in that they are weakly fluorescent both in their ON and OFF states. The possibility of photochromic switching without fluorescence in GFP-like proteins was recently predicted in a hypothetical photoswitching mechanism based on the chemistry of the model chromophore *p*-hydroxybenzylideneimidazolinone (HBI) [62]. The model proposes that coupling between protonation and bond isomerisation state in fluorescent protein chromophores is a natural consequence of the coupling between charge localisation and bond twisting in the excited state of the chromophore itself [62]. The mechanism (outlined Fig 5) helps to rationalise some properties of Phanta.

The model proposes there are *two* possible decay channels for the anionic chromophore, representing decay via twisted intramolecular charge-transfer (TICT) states corresponding to either of the two possible bridge double bonds. The two TICT states differ not only by twisted bond, but also by charge localisation. The imidazoloxy TICT channel features a twisted imidazoloxy bridge bond and a photoacidic excited state; the phenoxy TICT channel features a twisted phenoxy bond and a photobasic excited state. Reversible photoswitching pathways are achieved by coupling the neutral and anionic chromophores via nonadiabatic protonation/deprotonation in the imidazoloxy TICT channel facilitated by a reservoir of protons at a chemical potential intermediate between the pK_a_’s of the diabatic electronic states.

In the case of RSFPs, such as Dronpa and eCGP123, access to the phenoxy TICT channel for the anionic chromophore in the ON state is restricted by the protein matrix, thereby allowing radiative decay to predominate [62]. Decay via the phenoxy TICT channel should regenerate the ground state of the anion. If the restriction on access to the phenoxy channel in the ON state were lifted, a protein displaying photochromic switching without fluorescence switching would result. This is precisely the behaviour observed in Phanta, eCGP123^H193Q^ and some other eCGP123^H193X^ variants. Intensity-driven photoswitching from ON to OFF can be explained by a small activation barrier to access the imidazoloxy TICT channel in the excited state, while a phenoxy TICT channel (with smaller or no barrier) provides a mechanism for non-radiative regeneration of the ground state. Intensity independent OFF to ON switching by violet light can be explained by a barrierless entrance to the imidazoloxy TICT channel in the excited state of the neutral chromophore.

The structures of Phanta, eCGP123, eCGP123^H193Q^ and eCGP123^T69V^ show only modest variation in the vicinity of the chromophore. The most noticeable difference occurs in the conformation of residue Arg66, which is in contact with the chromophore (S5 Fig). This residue would usually be assumed cationic. It is possible that different placement of this charged residue modulates the accessibility of the TICT pathways in the different proteins. Further data is needed to seriously test this suggestion. The displacement of the arginine is also large for the Thr69Val mutant, which has significant fluorescence (Φ_F_, 0.32; Table 1).

The absorbance spectra of His193X substituted proteins suggest the partition of neutral and anionic chromophores species can be strongly influenced (Fig 1). Increased fluctuation in the proton chemical potential experienced by the phenoxy site could contribute to nonadiabatic transitions in the TICT channels. The His193 residue in the parent eCGP123 interacts via a *p*-stacking interaction with the phenoxy ring of the chromophore; it is conceivable that the orientational selectivity of this interaction may hinder torsional motion of the phenoxy ring. Both of these differences could conceivably contribute to altering the phenoxy TICT channel cross-section in His193 substitution mutants of eCGP123.

There has been active study of the photoswitching reaction in the fluorescent photoswitching proteins Dronpa and Dronpa2 (Dronpa^M159T^). These proteins have ON and OFF states with spectra similar to the photochromic protein Phanta. Photochromic switching as in Phanta is observed in Dronpa, with the main difference being that in Dronpa this is also accompanied by switching of the fluorescence yield. In the case of Dronpa and the M159T mutant, there has been significant debate about the identity of steps involved in the photoswitching reaction, and their ordering. Early results of transient absorption results following excitation of the OFF state of Dronpa were interpreted by Fron et al. as indicative of excited-state proton transfer preceeding isomerization during the photoreaction [63]. However, subsequent ultrafast vibrational spectroscopic studies by Warren et al. and Kaucikas et al. appeared to contraindicate this, and were taken to suggest deprotonation on the ground state following excitation of the OFF state to yield ON [64,65]. An independent ultrafast vibrational spectroscopy study by Lukacs et al. failed to find conclusive evidence of either photoisomerization or proton transfer in the excited state, and indicated ground-state depronation [66]. A broadband UV-vis transient absorption study by Yadav et al. reported direct observation of the photoisomerization reaction on a ps timescale, followed by ground-state deprotonation on the μs timescale [67]. Since the twisted states are predicted by theory to have twisted intra-molecular charge-transfer character, the electronic gap will be coupled to other modes in addition to the proton occupancy at the phenoxy site, so that the mechanism is simply modified to allow titration subsequent to the non-adiabatic event. The mechanism in Fig 5 allows for the existence of non-productive OFF-> ON switching pathways, such as are indicated for Dronpa by a recently revised quantum yield estimate of the photoreaction, which is lower than previous estimates [67]. Determination of the relative role of protonation and deprotonation in the photoswitching of Phanta and its variants requires ultrafast spectroscopy experiments such as have been reported for Dronpa and Dronpa2 [63–65,67].

In the case of the related, but different, green-to-red photoswitchable fluorescent proteins, a transient dark state has been suggested on the basis of simulations to exist, generated by protonation of the methine bridge of the chromophore, following intersystem crossing to the triplet state or from an alternate radical ground state [68]. If the bridge were to become transiently protonated then we would assume an *sp* ^*3*^ hybridization state with a reduced barrier for bridge rotation. In the case of the green-to-red photoactivatible proteins IrisFP, this was supported by crystallographic evidence of significant structural distortion local to the chromophore bridge [69].

We have yet to determine a crystal structure for Phanta in the OFF state. However, compared to Dronpa, the fluorescent eCGP123^T69V^ and eGP123 have a similar arrangement of amino acid side chains around the chromophore (S6 Fig) including a histidine at position 193 and are likely to contain *trans*-chromophores as observed in Dronpa. Nevertheless, this is not necessary, because nonadiabatic proton transfer in the imidazoloxy TICT does not necessarily need to subsequently produce a *trans*-photoproduct. The relevant twisted conical intersections are of charge-transfer character, and so their position in energy and in configuration space depends sensitively on the electrostatic environment of the chromophore in the pocket [70] and thereby the protonation state of residues in the protein interior, which cannot be directly determined by X-ray crystallography. The electrostatic fields on the interior of some fluorescent protein variants can range as high as 100 MV/cm [71,72]. This suggests that the range of interaction energies of the diabatic states with the local electrostatic field in the interior of fluorescent proteins could be sufficient to drive nonadiabatic electronic transitions in the chromophore TICT channels (or alternatively, to suppress them entirely).

## Conclusion

Phanta is a weakly photoswitching protein suitable for use as an acceptor for pcFRET. The data described here provide insights into the structural reasons for its very low Φ_F_. Non-coplanar chromophores or chromophores in alternate conformations and different protein matrix environments are reasons reported for low fluorescence efficiency in other proteins. In Phanta and eCGP123^H193Q^, the chromophore remains *cis-* and most likely coplanar in the ground state. The side chain of His193 is critical for efficient fluorescence emission. Its substitution results in the loss of a number of important stabilising interactions including π-π interactions and changes to the scaffold beneath the chromophore. Variants having substitutions at 193 with a small and aliphatic or small polar side chain favour the anionic chromophore, retain their ability to photoswitch, and may find use as pcFRET acceptors. A negatively charged side chain at position 193 favours the formation of a protonated chromophore, and although unable to photoswitch such variants might be useful as non-fluorescent FRET acceptors for the emissions of blue fluorescence proteins, such as Sirius (λ_max_^em^, 424 nm) [73]. The absorbance spectra of other non-fluorescent dark acceptors such as the REACh series of proteins [20,21] and Ultramarine [19] are red-shifted and unsuitable as acceptors for the emissions of blue fluorescent proteins. Finally, there is the possibility of engineering other Phanta-like proteins with different or improved properties useful for pcFRET. This might include proteins that normally reside in the OFF state, or those that exhibit bistable photoswitching behaviour.

## Supporting Information

S1 Fig The amino acid sequence of Phanta and selected fluorescent proteins. The amino acid sequence for Phanta is shown aligned with selected photoswitching fluorescent proteins including its highly fluorescent parent eCGP123. Phanta shows 77.7%, 74.2% and 77.3% amino acid identity with Dronpa, mEOS and Dendra2, respectively. The C-terminal His6 tag used for protein purification has been omitted from the sequence. Arrowheads indicate the three amino acid residues that differ between eCGP123 and Phanta. The chromophore tripeptide for each protein is shown highlighted by the yellow shading.
(TIF)

S2 Fig pH titration curves for eCPP123 and selected variants. Absorbance at two selected wavelengths are shown for each variant at different pH.
(TIF)

S3 Fig Photoswitching of eCGP123^H193Q^. (A) Relative absorbance is shown for eCGP123^H193Q^ on sequential illumination with cyan photoswitching light (open head arrow) and violet light (dashed arrow). (B) As in (A) but with a further period of incubation at 25°C without photoswitching illumination indicated by filled arrow head, followed by a further cycle of photoswitching initiated at 40 mins.
(TIF)

S4 Fig An alternate view of the Phanta chromophore environment. An alternate stereoview is shown for the chromophore environment and hydrogen bond network of Phanta that involves the sidechains of Arg91, Tyr 177 and O2 of the imidazalinone. Numbered waters are shown as red spheres.
(TIF)

S5 Fig The position of Arg66 in eCGP123 variants. The position of the Arg66 side chain is shown for eCGP123 (green), eCGP123^H193Q^ (magenta), Phanta (orange) and eCGP123^T69V^ (light blue) relative to the side chain at position 69 and 193, and the chromophore.
(TIF)

S6 Fig Amino acid side chains involved in the photoswitching of Dronpa compared with Phanta. Key side chains implicated in the photoswitching mechanism of Dronpa are shown. Side chains for the ON (fluorescent) state and OFF (non-fluorescent) state of Dronpa are shown in yellow and teal, respectively. Superposed are the equivalent side chains for Phanta in the ON (anionic) state. Isomerisation of the chromophore on photoswitching of Dronpa is accompanied by repositioning of Arg66 and His193 (Dronpa numbering). The coordinates 2IOV and 2POX were used to generate the visualisation of the Dronpa chromophore.
(TIF)

S1 Table Phanta chromophore contacts. (DOCX)

S2 Table eCGP123^T69V^ chromophore contacts. (DOCX)

S3 Table eCGP123^H193Q^ chromophore contacts. (DOCX)
