# Supplementary figures and images for: X-Ray Crystal Structure and Properties of Phanta, a Weakly Fluorescent Photochromic GFP-Like Protein
Source: PLoS One. 2015 Apr 29;10(4):e0123338. doi: 10.1371/journal.pone.0123338 (PMC4414407; doi:10.1371/journal.pone.0123338)

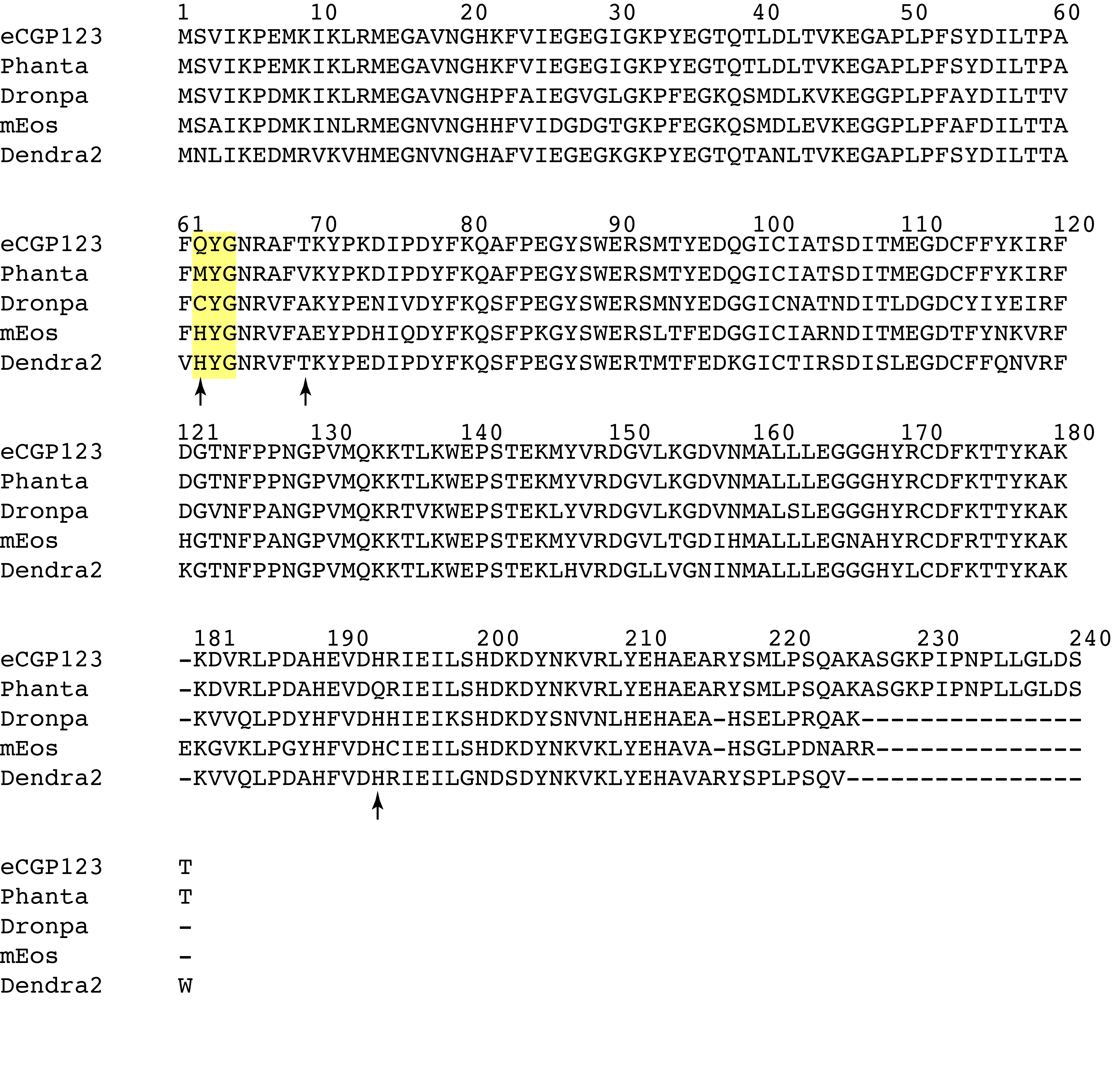

Supplement: S1 Fig — The amino acid sequence for Phanta is shown aligned with selected photoswitching fluorescent proteins including its highly fluorescent parent eCGP123. Phanta shows 77.7%, 74.2% and 77.3% amino acid identity with Dronpa, mEOS and Dendra2, respectively. The C-terminal His6 tag used for protein purification has been omitted from the sequence. Arrowheads indicate the three amino acid residues that differ between eCGP123 and Phanta. The chromophore tripeptide for each protein is shown highlighted by the yellow shading. (TIF) [file pone.0123338.s001.tif]

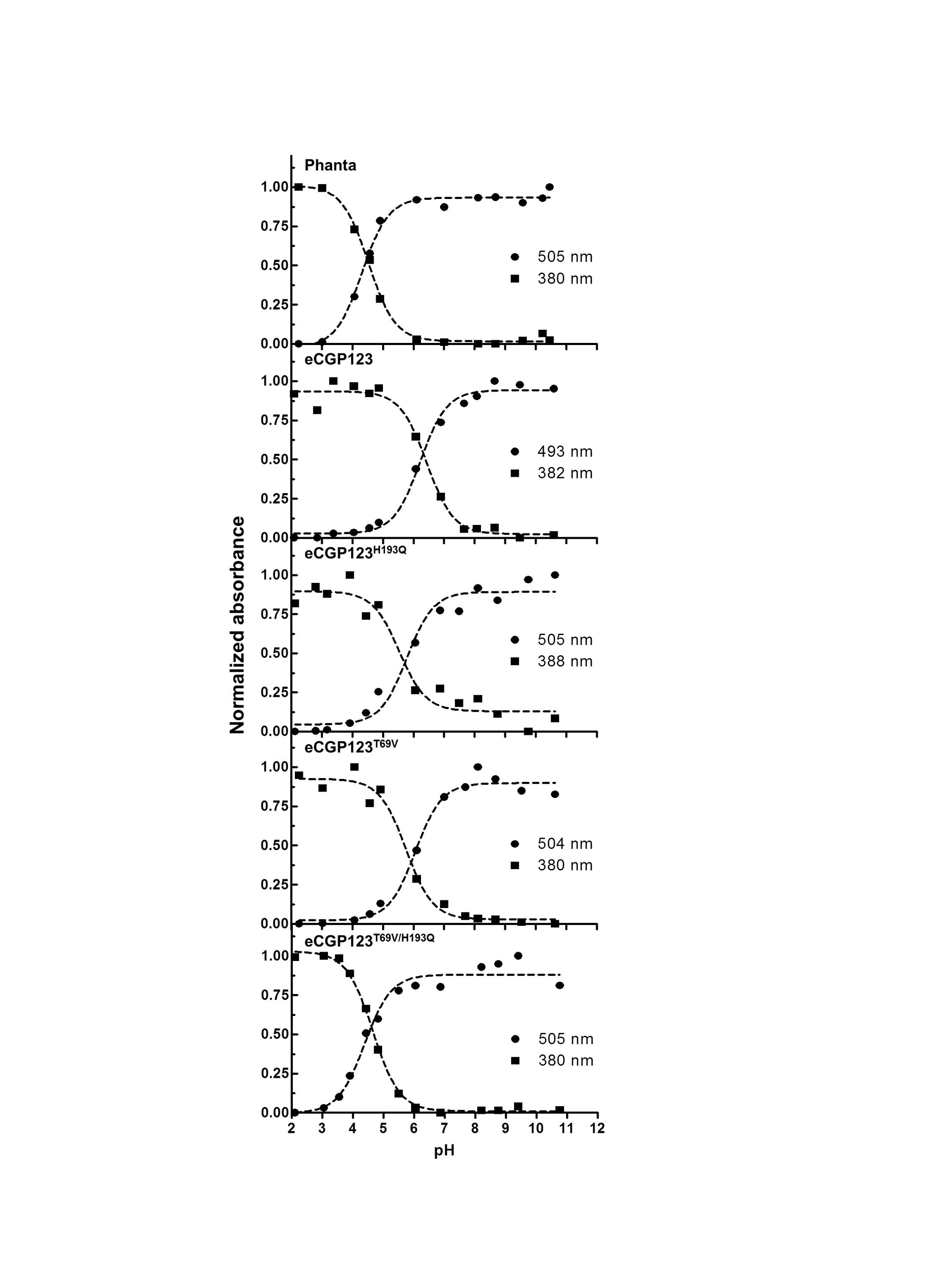

Supplement: S2 Fig — Absorbance at two selected wavelengths are shown for each variant at different pH. (TIF) [file pone.0123338.s002.TIF]

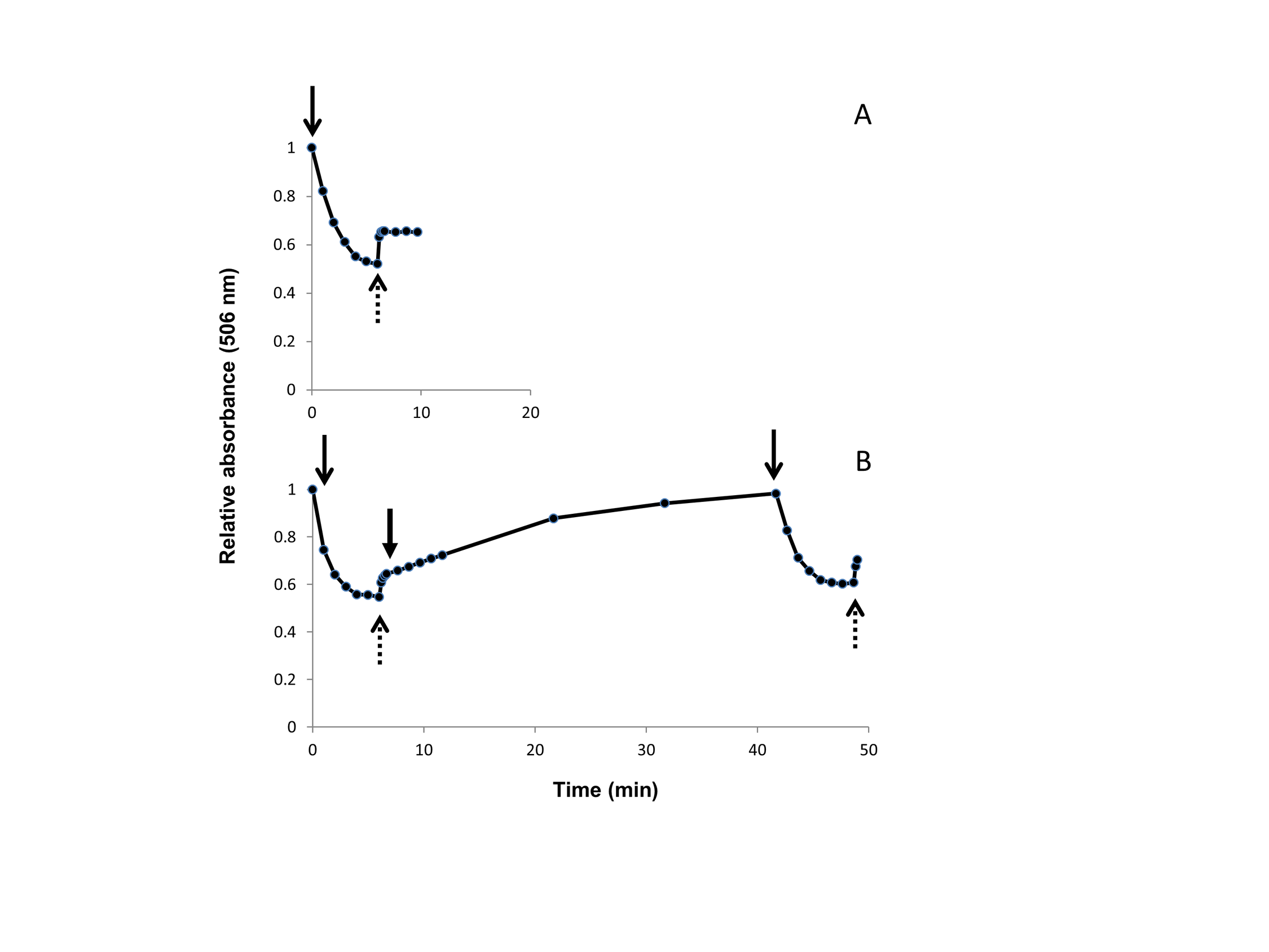

Supplement: S3 Fig — (A) Relative absorbance is shown for eCGP123H193Q on sequential illumination with cyan photoswitching light (open head arrow) and violet light (dashed arrow). (B) As in (A) but with a further period of incubation at 25°C without photoswitching illumination indicated by filled arrow head, followed by a further cycle of photoswitching initiated at 40 mins. (TIF) [file pone.0123338.s003.TIF]

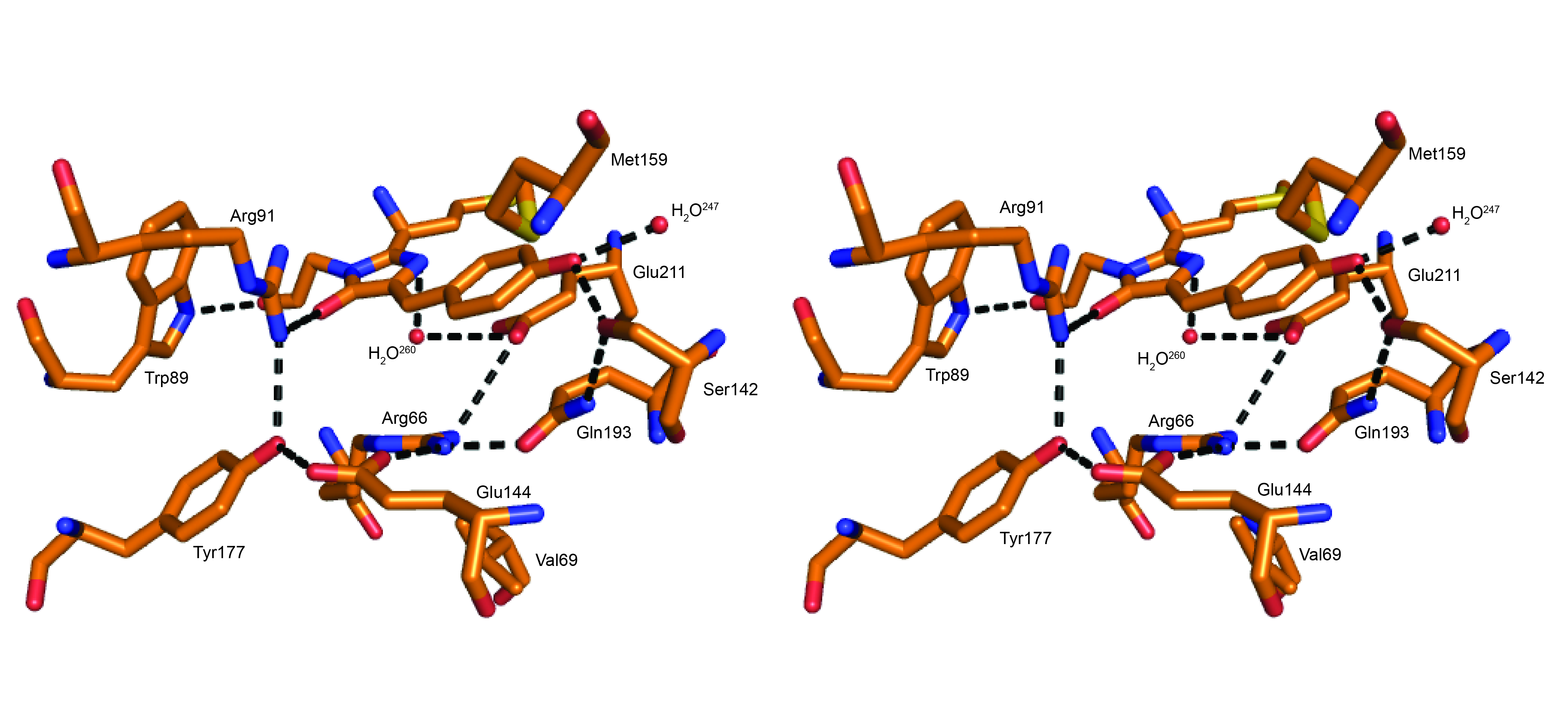

Supplement: S4 Fig — An alternate stereoview is shown for the chromophore environment and hydrogen bond network of Phanta that involves the sidechains of Arg91, Tyr 177 and O2 of the imidazalinone. Numbered waters are shown as red spheres. (TIF) [file pone.0123338.s004.tif]

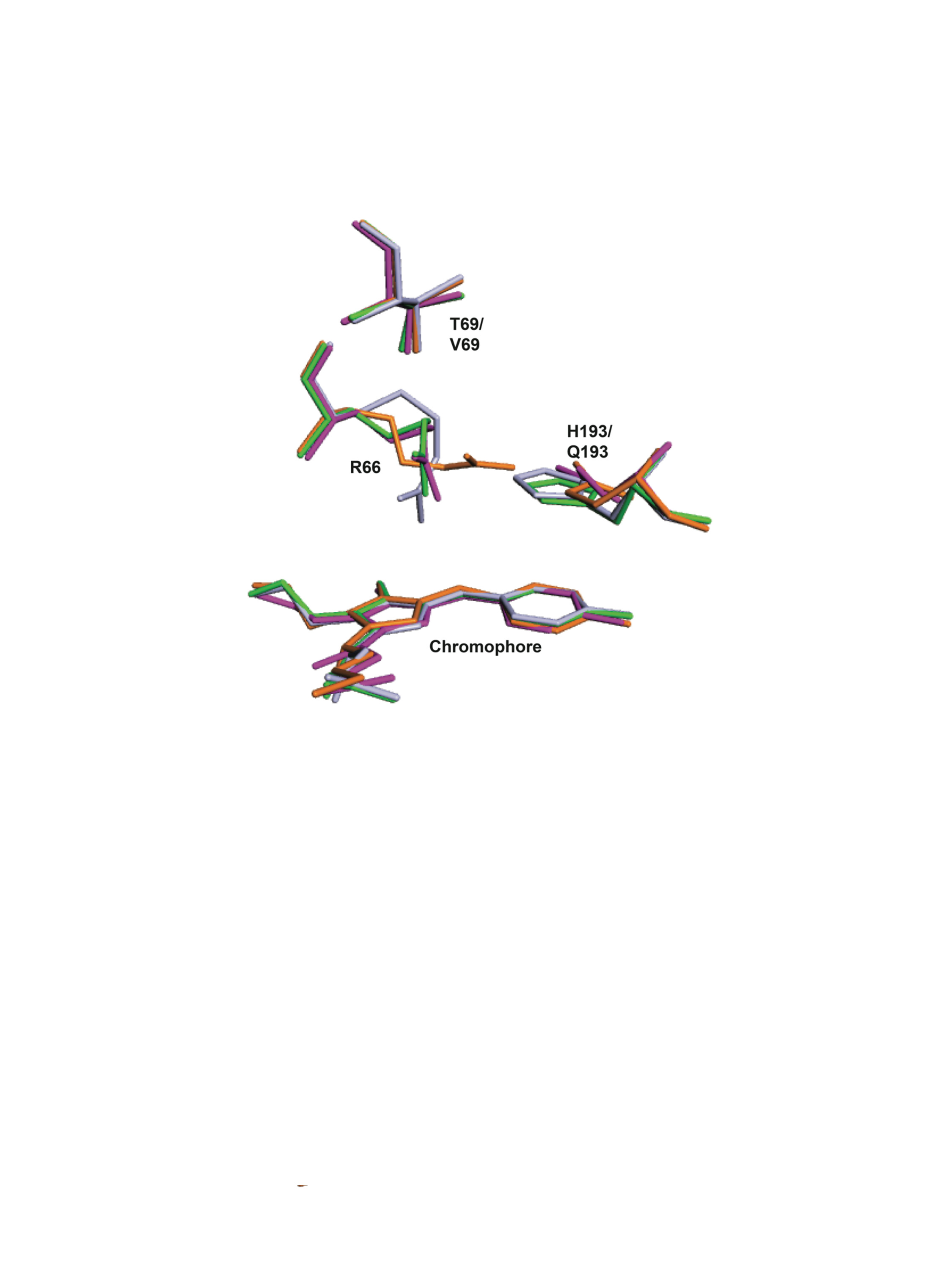

Supplement: S5 Fig — The position of the Arg66 side chain is shown for eCGP123 (green), eCGP123H193Q (magenta), Phanta (orange) and eCGP123T69V (light blue) relative to the side chain at position 69 and 193, and the chromophore. (TIF) [file pone.0123338.s005.TIF]

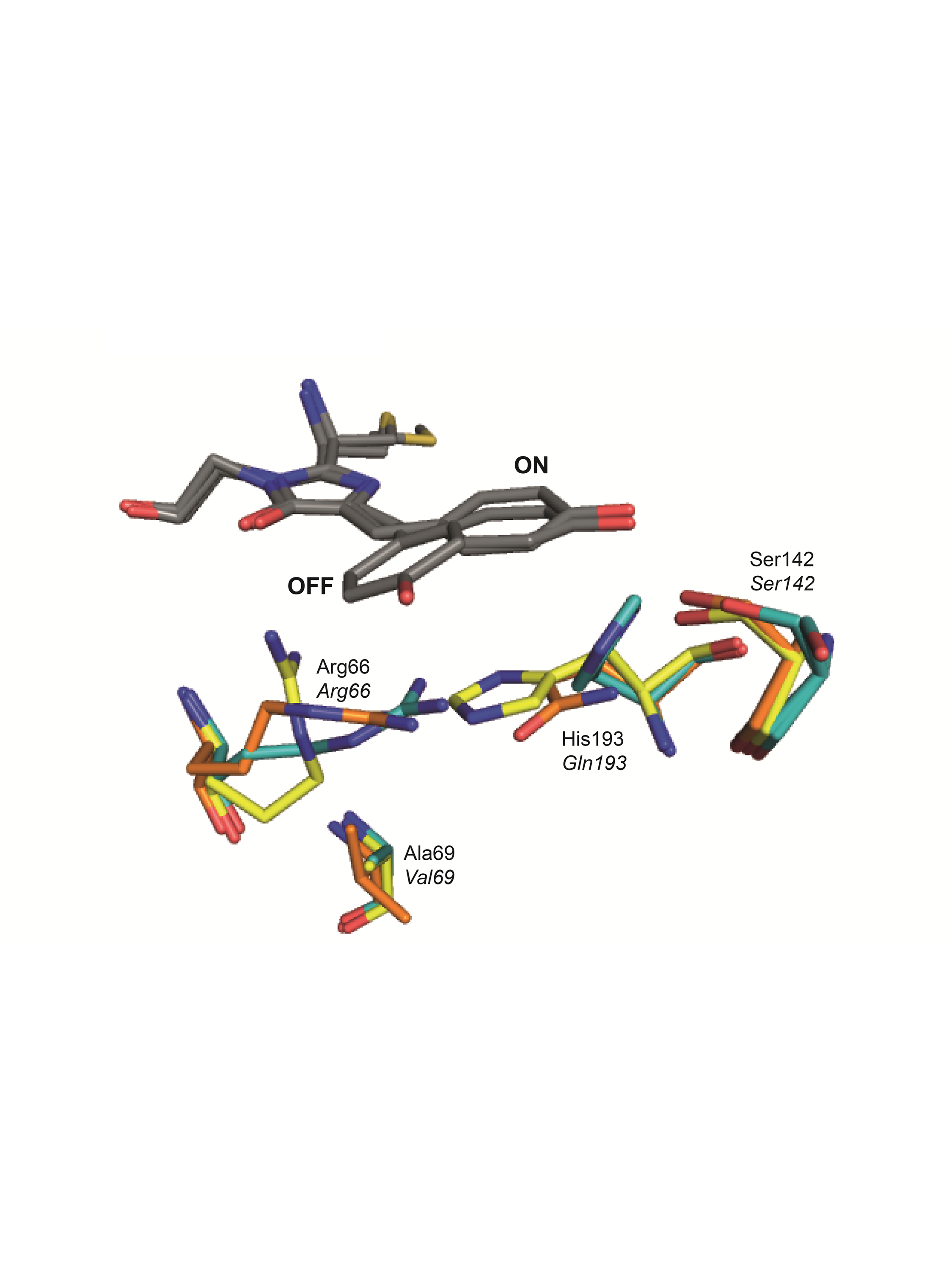

Supplement: S6 Fig — Key side chains implicated in the photoswitching mechanism of Dronpa are shown. Side chains for the ON (fluorescent) state and OFF (non-fluorescent) state of Dronpa are shown in yellow and teal, respectively. Superposed are the equivalent side chains for Phanta in the ON (anionic) state. Isomerisation of the chromophore on photoswitching of Dronpa is accompanied by repositioning of Arg66 and His193 (Dronpa numbering). The coordinates 2IOV and 2POX were used to generate the visualisation of the Dronpa chromophore. (TIF) [file pone.0123338.s006.TIF]
