# Supplementary material for: X-Ray Crystal Structure and Properties of Phanta, a Weakly Fluorescent Photochromic GFP-Like Protein
Source: PLoS One. 2015 Apr 29;10(4):e0123338. doi: 10.1371/journal.pone.0123338 (PMC4414407; doi:10.1371/journal.pone.0123338)
Supplement: S1 Table — (DOCX) [file pone.0123338.s007.docx]

**S1 Table.** Phanta chromophore contacts

| **Chromophore atom** | **Interacting protein atom(s)** | **Nature of interaction(s)** |
| --- | --- | --- |
| **Methionine moiety** | | |
| N1 | Ala60 O, Thr58 O | H-bond |
|  | Ala60 C | vdw |
| C^α1^ | Asn65 N | vdw |
| C^β1^ | Thr58 O | vdw |
| C^γ1^ | Gln38 O^ε1^/C^δ^/N^ε2^ | vdw |
| S1 | Gln38 O^ε1^, Glu211 N | H-bond |
| C1 | Leu209 O, Gln38 C^δ^/C^γ^ | vdw |
| **Imidazolinone moiety** | | |
| C4 | Pro59 O, Asn65 N | vdw |
| N2 | Pro59 O, Glu211 O^ε1^/O^ε2^ | H-bond |
|  | Pro59 C^α^, Glu211 C^δ^ | vdw |
|  | Glu211 O^ε1^, Gln38 N^ε2^, Asn65 O/N | H_2_O^(260)^ mediated H-bond |
| C^α2^ | Pro58 C^α^, Glu211 C^δ^ | vdw |
| C2 | Pro59 O/C^2^, Arg91 C^ζ^/N^H1/ H2^, Asn64 N | vdw |
| O2 | Arg91 N^H1^/N^H2^, Pro59 O, Asn64 N | H-bond |
|  | Arg91 C^ζ^ | vdw |
|  | Trp89 N^ε1^, Asn65 N, Arg66 N | H_2_O^(123)^ mediated H-bond |
| N3 | Asn69 N, Pro63 O | H-bond |
|  | Glu211 O^ε1^, Gln38 N^ε2^, Asn65 O/N | H_2_O^(260)^ mediated H-bond |
| **Glycyl moiety** | | |
| C^α3^ | Asn65 N, Ala60 O | vdw |
| C3 | Asn65 C^3^, Trp89 N^ε1^ | vdw |
| O3 | Trp89 N^ε1^ | H-bond |
|  | Trp89 N^ε1^, Asn65 N, Arg66 N | H_2_O^(123)^ mediated H-bond |
|  | Ser105 O^γ^, Ala60 O | H_2_O^(257)^ mediated H-bond |
| **4-hydroxyphenyl-methylene moiety** | | |
| C^β2^ | Glu221 O^ε2^, Arg66 C^z^ | vdw |
| C^γ2^ | Glu221 O^ε2^ | vdw |
| C^δ1^ | Glu221 O^ε2^, Pro59 C^β^ | vdw |
| C^δ2^ | – |  |
| C^ε1^ | Ile195 C^ε1/Cγ2^ | vdw |
| C^ε2^ | Gln193 C^δ^, Met159 S^δ^/C^ε^ | vdw |
| C^ζ^ | Gln193 C^γ^, Met159 S^δ^/C^ε^, Ser142 O^γ^ | vdw |
| O^H^ | Met159 S^δ^/C^ε^ | vdw |
|  | Ser142 O^γ^ | H-bond |
|  | Glu140 O | H_2_O^(247)^ mediated H-bond |
