# Supplementary material for: X-Ray Crystal Structure and Properties of Phanta, a Weakly Fluorescent Photochromic GFP-Like Protein
Source: PLoS One. 2015 Apr 29;10(4):e0123338. doi: 10.1371/journal.pone.0123338 (PMC4414407; doi:10.1371/journal.pone.0123338)
Supplement: S2 Table — (DOCX) [file pone.0123338.s008.docx]

**S2 Table. e**CGP123^T69V^ chromophore contacts

| **Chromophore atom** | **Interacting protein atom(s)** | **Nature of interaction(s)** |
| --- | --- | --- |
| **Glutamate moiety** | | |
| N | Ala60 O, Pro59 O, Thr58 O | H-bond |
|  | Pro59 C, Ala60 C, Phe61 C^α^, | vdw |
| C^α1^ | Pro59 O, Glu211 O^ε1^, Thr58 O | vdw |
| C^β1^ | Glu211 O^ε1^/C^β^, Gln38 C^δ^/N^ε2^ | vdw |
| C^γ1^ | Leu40 C^δ1^, Gln38 C^δ^/N^ε2^/O^ε1^ | vdw |
| C^δ3^ | Gln38 O^ε1^/C^δ^, Leu209 C^γ^, Glu211 C^β^/N | vdw |
| N^ε1^ | Gln38 O^ε1^, Leu209 O | H-bonds |
|  | Gln38 C^δ^/C^γ^, Leu2 C^δ1^, Tyr210 C | vdw |
| O^ε1^ | Glu211 N, Tyr210 O/N | H-bonds |
|  | Ile195 C^γ1^/C^δ1^, Glu211 C^α^/C^β^, Tyr210 C/C^α^, Leu209 C^γ^/C^δ1^/C^δ2^ | vdw |
| **Imidazolinone moiety** | | |
| C1 | Asn65 N,Glu211 O^ε1^ | vdw |
| N2 | Pro59 O, Glu211 O^ε1^ | H-bonds |
|  | Pro59 C/C^α^, Glu211 C^δ^ | vdw |
|  | Asn65 O/N, Glu211 O^ε1^, Gln38 N^ε2^, Arg66 N^H2^ | H_2_O^(10)^ mediated H-bond |
| C^α2^ | Arg66 N^H1^/N^H2^/C^ζ^, Pro59 C^α^/C/O | vdw |
| C2 | Arg66 N^H1^/N^H2^/C^ζ^, Pro59C/O, Arg91 N^H1^/N^H2^ | vdw |
| O2 | Arg66 N^H1^/N^H2^, Arg91 N^H1^/N^H2^, Pro59 O | H-bonds |
|  | Arg66 C^ζ^, Arg91 C^ζ^, | vdw |
|  | Trp89 N^ε1^, Arg66 N/N^H1^/N^H2^, Asn65 N | H_2_O^(144)^ mediated H-bond |
| N3 | Asn65 N, Pro59 O | H-bonds |
|  | Pro59 C | vdw |
|  | Asn65 O/N, Glu211 O^ε1^, Gln38 N^ε2^, Arg66 N^H2^ | H_2_O^(10)^ mediated H-bond |
| **Glycyl moiety** | | |
| C^α3^ | Asn65 N/C^α^, Ala60 C/O, Pro59 O, Phe61 C/O | vdw |
| C | Asn65 C/C^α^/C^β^, Trp89 N^ε2^ | vdw |
| O | Trp89 N^ε1^, Asn65 N | H-bond |
|  | Ile107 C^δ1^, Asn61 C^α^/C^β^ | vdw |
|  | Ala61 O, Ser105 O^γ^ | H_2_O^(184)^ mediated H-bond |
|  | Trp89 N^ε1^, Arg66 N/N^H1^/N^H2^, Asn65 N | H_2_O^(144)^ mediated H-bond |
| **4-hydroxyphenyl-methylene moiety** | | |
| C^β2^ | Arg66 N^H1^/N^H2^/C^ς^, Pro59 O | vdw |
| C^γ2^ | His193 N^ε1^/C^ε1^ | vdw |
| C^δ1^ | His193 N^ε2^/N^δ1^/C^γ^/C^ε1^/C^δ2^ | vdw |
| C^δ2^ | His193 N^δ1^, Pro59 C^β^, Glu211 O^ε2^/C^δ^ | vdw |
| C^ε1^ | Ser142 O^γ^, His193 N^δ1^/C^γ^/C^δ2^, Met159 S^δ^/C^ε^ | vdw |
| C^ε2^ | His193 N^δ1^/C^γ^, Ile195 C^β^/C^γ2^, Met159 S^δ^/C^ε^ | vdw |
| C^ς^ | Ser142 O^γ^, His193 C^β^/C^γ^, Met159 S^δ^/C^ε^ | vdw |
| O^H^ | Ser142 O^γ^ | H-bond |
|  | Ser142 C^β^, His193 C^β^, Met159 S^δ^/C^ε^ | vdw |
|  | Ile195 N, His193 O, Glu140 O, Arg194 N, Ser142 N | H_2_O^(238)^ mediated H-bond |
