# Supplementary material for: X-Ray Crystal Structure and Properties of Phanta, a Weakly Fluorescent Photochromic GFP-Like Protein
Source: PLoS One. 2015 Apr 29;10(4):e0123338. doi: 10.1371/journal.pone.0123338 (PMC4414407; doi:10.1371/journal.pone.0123338)
Supplement: S3 Table — (DOCX) [file pone.0123338.s009.docx]

**S3 Table.** eCGP123^H193Q^ chromophore contacts

| **Chromophore atom** | **Interacting protein atom(s)** | **Nature of interaction(s)** |
| --- | --- | --- |
| **Glutamate moiety** | | |
| N | Phe15 O, Ala60 O | H-bond |
|  | Phe61 C^α^ | vdw |
|  | Phe61 O, Tyr116 O^H^, Gln42 O^ε1^/N^ε1^ | H_2_O^(34)^ mediated H-bond |
| C^α1^ | Phe61 C, Pro59 O, Glu211 O^ε1^, Gln38 N^ε2^, | vdw |
| C^β1^ | Glu211 O^ε1^/C^β^, Gln38 N^ε1^, Ile195 C^δ1^, Phe61 C | vdw |
| C^γ1^ | Phe61 C, Leu209 C^δ2^, Gln38 C^δ^/N^ε^/O^ε1^ | vdw |
| C^δ3^ | Gln38 O^ε1^/N^ε1^/C^δ^, Leu209 C^γ^/C^δ2^, Glu211 C^β^ | vdw |
| N^ε1^ | Gln38 O^ε1^, Leu209 O | H-bond |
|  | Gln38 C^δ^/ C^γ^, Leu40 C^δ1^/C^β^, Leu209 C/C^γ^/C^δ2^, | vdw |
| O^ε1^ | Tyr210 N, Glu211 N | H-bond |
|  | Leu209 C/C^γ^, Tyr210 C, Glu211 C^α^/C^β^/C^γ^, Ile195 C^γ1^ | vdw |
| **Imidazolinone moiety** | | |
| C1 | Pro59 O/C, Asn65 N,Glu211 O^ε1^ | vdw |
| N2 | Glu211 O^ε1/ ε2^, Pro59 O | H-bonds |
|  | Glu211 C^δ^, Pro59 C/C^α^ | vdw |
|  | Glu211 O^ε1/ ε2^, Gln38 N^ε2^, Asn65 N/O, Arg66 N^H1^/N^H2^ | H_2_O^(35)^ mediated H-bond |
| C^α2^ | Arg70 N^H1^, Arg95 N^H1^, Pro63 O/C/C^α^ | vdw |
| C2 | Arg66 N^H1^, Arg91 N^H1^/N^H2^, Pro59 C/O, Asn65 N | vdw |
| O2 | Arg91 N^H1^/N^H2^, Pro59 O, Asn65 N | H-bond |
|  | Arg91 C^ξ^ | vdw |
|  | Tyr87 O^H^, Trp89 N^ε1^, Arg6 N, Asn65 N | H_2_O^(785)^ mediated H-bond |
| N3 | Asn6 N, Pro59 O | H-bonds |
|  | Phe61 C, Pro59 C | vdw |
|  | Glu211 O^ε1/ ε2^, Gln38 N^ε2^, Asn65 N/O, Arg66 N^H1^/N^H2^ | H_2_O^(35)^ mediated H-bond |
| **Glycyl moiety** | | |
| C^α3^ | Asn65 C^α^/N, Ala60 O, Phe61 C | vdw |
| C | Asn65 C/C^α^/C^β^, Trp89 N^ε1^ | vdw |
| O | Trp89 N^ε1^ | H-bond |
|  | Asn65 C^α^/C^β^, Ile107 C^δ1^, Trp89 C^ε2^/C^ζ2^ | vdw |
|  | Tyr87 O^H^, Trp89 N^ε1^, Arg66 N, Asn65 N | H_2_O^(785)^ mediated H-bond |
| **4-hydroxyphenyl-methylene moiety** | | |
| C^β2^ | Arg66 N^H1^, Arg91 N^H1^/N^H2^, Pro59 C^β^/O | vdw |
| C^γ2^ | Arg66 N^H1^, Pro59 C^β^ | vdw |
| C^δ1^ | Glu211 O^ε2^, Pro59 C^α^/C^β^ | vdw |
| C^δ2^ | Gln193 C^δ^ | vdw |
| C^ε1^ | Met159 C^ε^, Ile195C^β^/C^γ2^ | vdw |
| C^ε2^ | Met159 C^ε^/S^δ^, Ser142 O^γ^, Gln193 C^δ^/N^ε2^ | vdw |
| C^ς^ | Ser142 O^γ^, Gln193 C^β^/N^ε2^, Met159 C^ε^/S^δ^ | vdw |
| O^H^ | Ser142 O^γ^ | H-bond |
|  | Ser142 C^β^, Gln193 C^β^, Met159 S^δ^/C^ε^, Ile195 C^γ2^ | vdw |
|  | Glu140 O, Ile195 N, Ser142 O^γ^ | H_2_O^(574)^ mediated H-bond |
